# Proceedings of ICIS SGCR-WIRES 2024, held jointly with the 23rd International Cancer Imaging Society Annual Conference, collaborating with the Singapore Radiological Society and College of Radiologists Singapore

**DOI:** 10.1186/s40644-024-00751-2

**Published:** 2024-09-12

## Oral presentations

### O1 A randomized controlled trial of preoperative prostate artery embolization before transurethral resection of prostate glands larger than 80cc

#### Zong Yi Chin^1^, Alvin YM Lee^2^, Neo Shu Hui^3^, Ng Tze Kiat^2^, Edwin Jonathan Aslim^2^, Allen SP Sim^4^, Pradesh Kumar^5^, John SP Yuen^2^, Kenneth Chen^2^, Sivanathan Chandramohan^1^

##### ^1^Vascular and Interventional Radiology, Singapore General Hospital, Singapore; ^2^Urology, Singapore General Hospital, Singapore; ^3^Urology, Sengkang General Hospital, Singapore; ^4^Urology, Gleneagles Medini Johor, Malaysia; ^5^Radiology, Sunway Medical Centre, Malaysia

*Cancer Imaging (2024)*, **24 (1):** O1

**Objectives/ Teaching Points:**

To study the impact of preoperative prostate artery embolization (PAE) on intraoperative blood loss during transurethral resection of the prostate (TURP) for large prostates (exceeding 80 cc).

**Material(s) and Method(s):**

A prospective, surgeon-blinded, randomized controlled trial was performed at a single tertiary centre. Patients with prostate volumes over 80 cc who needed TURP were randomly allocated (1:1) to receive preoperative prostatic artery embolization followed by TURP (Group A—intervention) or TURP alone (Group B—control). The primary outcome measured the postoperative drop in haemoglobin levels, as a surrogate for blood loss. Secondary outcomes studied included resection efficiency (weight of resected tissue per minute) and the rate of postoperative complications.

**Results:**

There were 10 patients in each group. The median prostate volumes were 119 mL for Group A and 140 mL for Group B, with median preoperative haemoglobin levels of 13.3 g/dL (IQR 12.5–14.3 g/dL) in Group A and 14.4 g/dL (IQR 10.1–15.2 g/dL) in Group B. The decrease in postoperative haemoglobin was significantly greater in Group B compared to Group A (-1.4 g/dL vs + 0.5 g/dL, p = 0.015). There were no significant differences between the groups in terms of the weight of resected prostate tissue (52 g vs 73 g, p = 0.089) and resection efficiency (0.7 g/min vs 0.6 g/min, p = 0.853). Two patients in Group B needed a red blood cell transfusion, compared to one patient in Group A (p = 1.000). One patient from each group required an additional surgery for haemostasis.

**Conclusions:**

Preoperative PAE significantly decreased TURP blood loss in men with large prostates.

### O2 Improving AI Transparency Using an Uncertainty-inspired Classification Model for Chest Xray Diagnosis

#### Shu Wen Goh^1^, Yangqin Feng^2^, Xinxing Xu^2^, Yong Liu^2^, Cher Heng Tan^1^

##### ^1^Diagnostic Radiology, Tan Tock Seng Hospital, Singapore; ^2^Institute of High-Performance Computing, A*STAR, Singapore

*Cancer Imaging (2024),* **24 (1):** O2

**Objectives/ Teaching Points:**

Most radiology artificial intelligence (AI) models are designed to recognise a predefined set of diagnoses. However, real-world data is often more variable (e.g. rare conditions) and beyond the pre-trained set. We aim to address this by applying an uncertainty classification on a chest X-ray (CXR) AI model that calculates an uncertainty score to express its confidence in its prediction.

**Material(s) and Method(s):**

We trained a model for the probability of uncertainty across five specified categories (Cardiomegaly, Pneumonia, Pneumothorax, Tuberculosis, and Normal) and an associated uncertainty score (θ). A low (θ) indicates a confident inference, suggesting a reliable prediction within the five categories. A high (θ) indicates an image with ambiguous features or potentially a diagnosis that falls outside the predefined categories.

The model was trained on a dataset of 500 CXRs comprising the five categories, each containing 100 images. For each category, the data was partitioned into training and testing subsets with an 80:20 split.

**Results:**

Three models were trained and evaluated: DenseNet169, DenseNet169 with Stochastic Weight Averaging Densely (DenseNet169 + SWAD), and Uncertainty-inspired Open Set (UIOS). Their performance was assessed using the Area Under the Curve (AUC) of the Receiver Operator Characteristic. The DenseNet169 model achieved an AUC of 0.9767, the DenseNet169 + SWAD model had an AUC of 0.9805, and the UIOS model had an AUC of 0.9767.

**Conclusions:**

This study demonstrates a promising alternative indicator for diagnostic AI for conditions outside of trained diagnoses, with all three models showing a high classification accuracy. Incorporating uncertain probability increases the transparency and explainability of AI-driven diagnostic tools by identifying instances where predictions are likely to be unreliable and can prompt dedicated review by a radiologist.

### O3 Privacy preserving large language model for musculoskeletal MRI request form and protocoling augmentation: A feasibility study

#### James Thomas Patrick Decourcy Hallinan^1^, Naomi Wenxin Leow^2^, Yi Xian Low^1^, Aric Lee^1^, Wilson Ong^1^, Matthew Ding Zhou Chan^1^, Ganakirthana Kalpenya Devi^1^, Stephanie Shengjie He^1^, Daniel De-Liang Loh^1^, Desmond Shi Wei Lim^1^, Xi Zhen Low^1^, Ee Chin Teo^1^, Shaheryar Mohammad Furqan^3^, Tham Wei Yang Wilson^4^, Jiong Hao Tan^4^, Naresh Kumar^4^, Andrew Makmur^1^, Ting Yonghan^1^

##### ^1^Department of Diagnostic Imaging, National University Hospital, Singapore; ^2^AIO Innovation Office, National University Health System, Singapore; ^3^Division of Biomedical Informatics, Department from Surgery, Yong Loo Lin School of Medicine, National University of Singapore, Singapore; ^4^University Spine Centre, University Orthopaedics, Hand and Reconstructive Microsurgery (UOHC), National University Health System, Singapore

*Cancer Imaging (2024),* **24 (1):**O3

**Objectives/ Teaching Points:**

Utilise a privacy-preserving large language model (PP-LLM) to improve the clinical information on musculoskeletal MRI radiology request forms (RRFs) and automate protocoling.

**Material(s) and Method(s):**

In this retrospective study, musculoskeletal MRI RRFs randomly collected from June 2023-December 2023 were included. Studies without electronic medical record (EMR) entries were excluded. An institutional PP-LLM (Claude 2.0) was used to augment the original RRF by mining the EMR. The PP-LLM also suggested the appropriate protocol using the institutional guidelines. To assess PP-LLM consistency, each case was run six-times. Clinical information on the original and PP-LLM RRFs were compared using the Reason for exam Imaging Reporting and Data System(RI-RADS) by two musculoskeletal radiologists independently (Rad1;12-years-experience, Rad2:10-years-experience). These radiologists provided a consensus reference standard for protocoling, which was used for comparison against the PP-LLM and third-year in-training radiologists (RIT1 and 2). Overall percentage agreement (95%CIs) were calculated for RI-RADS gradings and protocol performance.

**Results:**

Overall, 500 musculoskeletal MRI RRFs were analysed (407 patients, mean age = 50.3 years ± 19.5[SD];202 women) across a range of cases; spine/pelvis (n = 143/500,28.6%), upper (n = 169/500,33.8%) and lower (n = 188/500,37.6%) extremities, and 170/500 (34%) required contrast. Clinical information for the PP-LLM RRFs was rated superior to the original request with 2.4-3.6% versus 12.4-22.6% rated as limited or deficient (RI-RADS C/D), respectively (p < 0.001). Protocoling accuracy for the PP-LLM was 85.0% (95%CI:81.9–88.1%), which was lower than RIT1 (91.8%;95%CI:89.4–94.2%) and RIT2 (90.8%;95%CI:88.3–93.3) (p < 0.001). Mean time for protocoling each study was slightly reduced for the PP-LLM (mean:13-14 s) compared to the radiologists in-training (mean:15-18 s). The PP-LLM had a high consistency with 440/500 cases (88%) having 100% (6/6 runs) agreement.

**Conclusions:**

Musculoskeletal MRI RRF augmentation by a privacy-preserving LLM provided superior clinical information to the clinician request, thereby potentially assisting radiologists with non-interpretive clinical tasks. The PP-LLM exhibited a high degree of accuracy and consistency in suggesting appropriate MRI protocols, and could relieve the burden of manual protocoling by radiologists.

### O4 Precision, Non-classifications, and Misclassifications of Large Language Models in Liver Lesions Classification using LI-RADS from Radiology Reports

#### Wan Hang Keith Chiu^1^, Jianliang Lu^2^, Ho-Ming Cheng^2^, Philip Leung-Ho Yu^3^, Wai-Kay Seto^2^

##### ^1^Department of Radiology and Imaging, Queen Elizabeth Hospital, Hong Kong; ^2^Department of Medicine, The University of Hong Kong, Hong Kong; ^3^Department of Mathematics and Information Technology, The Education University of Hong Kong, Hong Kong

*Cancer Imaging (2024),* **24 (1):** O4

**Objectives/ Teaching Points:**

Large Language Models (LLM) is a powerful tool for data extraction and summarization. However, scant evidence exists as to whether a task-specific model is necessary in medical applications. This study evaluates the performance of a general-purpose and a medically trained LLM in extracting and categorizing liver lesions from unstructured radiology reports according to the LI-RADS.

**Material(s) and Method(s):**

Sixty anonymized Computed Tomography (CT) reports, detailing 112 liver lesions from 5 institutions were fed into both GPT-4 and MedLM. Initially, simple prompts were used to assign LI-RADS categories (GPT4sp and MedLMsp). This was followed by detailed instructions post-prompt engineering (GPT4pe and MedLMpe). Reports were inputted once more to gauge reproducibility. Classification results derived by consensus from 2 radiologists served as ground truths. The accuracies between various techniques and LLMs were compared.

**Results:**

The accuracy rates for classifying a malignant lesion (LR-4/5/M) were 0.768, 0.759, 0.679, and 0.893 for GPT-4sp, MedLMsp, GPT-4pe, and MedLMpe respectively, with MedLM outperforming GPT-4 (p < 0.001, MedLMpe vs GPT-4pe). At patient level, performance was comparable, with accuracies of 0.833, 0.783, 0.783, and 0.883 for GPT4sp, MedLMsp, GPT-4pe, and MedLMpe, respectively (all p > 0.05). Prompt engineering improved performance (0.759 vs 0.893, p = 0.002) and reduced non-classification (2.7% vs 15.2%, p < 0.001) in MedLM but not in GPT-4 (0.768 vs 0.679, p = 0.123, and 22.3% vs 18.7% p = 0.452, respectively) and necessitated more resources than simple prompting (tokens used mean ± SEM—805 ± 191 vs 663 ± 217 p < 0.01 GPT4pe vs GPT4sp). MedLMpe had less misclassification (8.3% vs 12.6%, p =) and higher reproducibility (92.9% vs 80.4%, p = 0.006) than GPT4pe.

**Conclusions:**

While general-purpose LLM exhibits potential in text-based medical tasks, our findings suggest that medically trained LLM yields superior performance. Judicious application of prompt engineering could further enhance the quality of AI-generated results albeit at a higher cost.

### O5 CT-Based Body Composition Analysis in Survival Prediction for Ovarian Cancer Patients

#### Jih-An Cheng, Yu-Ching Lin, Yu-Fu Wu, Yen-Ling Huang, Keuian Chen, Yenpo Lin, Gigin Lin

##### Department of Medical Imaging and Intervention, Linkou Chang Gung Memorial Hospital, Taiwan

*Cancer Imaging (2024),* **24 (1):** O5

**Objectives/ Teaching Points:**

The purpose of this study was to evaluate the relationship of paraspinal muscle and fat composition (as determined by changes in attenuation and area of muscle, visceral and subcutaneous fat on CT) of patients with ovarian cancer to their overall and progression free survival.

**Material(s) and Method(s):**

This is a multi-institutional and retrospective study conducted from 2009 to 2018. Patients with ovarian cancer who underwent CT at the time of diagnosis were included. Patients were followed for at least 5 years after diagnosis or until death, whichever occurred first. Total measurements of psoas muscle, skeletal muscle, covering cross-sectional area and mean pixel intensity, alongside with the evaluation of fat compositions (including subcutaneous and visceral) at L4 vertebral level were obtained. Survival associations with changes in muscle measurements and fat compositions were evaluated using uni-variate and multivariate stepwise Cox proportional hazard analyses. All p-values were considered significant < 0.05.

**Results:**

A total of 496 patients were included (mean age ± SD, 49.3 ± 15.3 years; pT1 n = 233, pT2 n = 42, pT3 n = 221). Using the stepwise analysis, the subcutaneous fat mean density, pathological T and M stage, and age, were independently predictive for overall survival (hazard ratios (HR) 1.028, 6.102, 1.776 and 1.026, all p < 0.05), when normalized with pathological stage, histopathological types, age and status of resection margin. In subgroup analysis, the subcutaneous fat mean density remained an independent predictor for overall survival, in both early (pT1 and pT2) and late stage (pT3) ovarian cancers.

**Conclusions:**

Changes in subcutaneous fat composition upon the diagnosis of ovarian cancer could be an independent prognostic indicator for overall survival.

### O6 AI-powered automated tool for delineation of focal bone lesions and measurement of quantitative imaging biomarkers in Multiple Myeloma patients using Whole-Body Diffusion Weighted Imaging (WBMRI)

#### Antonio Candito^1^, Matthew D. Blackledge^2^, Richard Holbrey^2^, William Weston^3^, Francesca Castagnoli^3^, Alina Dragan^3^, Christina Messiou^3^, Dow-Mu Koh^3^

##### ^1^MRI Physics, The Institute of Cancer Research, United Kingdom; ^2^The Institute of Cancer Research, United Kingdom; ^3^The Royal Marsden NHS Foundation Trust, United Kingdom

*Cancer Imaging (2024),* **24 (1):** O6

**Objectives/ Teaching Points:**

WBMRI is recognised as the most sensitive imaging modality for detection of focal myeloma bone marrow lesions which are an integral part of diagnostic criteria. We have developed an automated tool to localise and delineate focal bone lesions from WBMRI which enables calculation of the Total Diffusion Volume (TDV, in millilitres) and the Apparent Diffusion Coefficient (ADC).

**Material(s) and Method(s):**

We used two retrospective, anonymised cohorts: a multi-centre cohort with 650 WBMRI scans from patients with multifocal and metastatic diseases, and a single-centre cohort with 66 patients diagnosed with multiple myeloma. Experienced radiologists manually delineated focal bone lesions in all 66 WBMRI scans.

The automated tool consists of five stages: (i) pre-processing raw DICOM images to compute b-value images and the ADC map; (ii) generating a signal b900 normalised image and a skeleton probability atlas map using a semi-supervised deep learning model developed from the multi-centre cohort; (iii) generating a mask of restricted diffusion bone lesions (high b900 signal and ADC > 600 mm /s) using a supervised shallow network trained on the single-centre cohort and images from (ii); (iv) excluding treated lesions (ADC > 1300 mm /s) using a post-processing algorithm; (v) generating a structured report with TDV and ADC measurements from the delineated regions.

**Results:**

The automated tool achieves an average dice score of 0.61 and a precision of 0.73 per scan compared to manual expert delineations on 13 test datasets. The relative differences in median ADC and TDV (log-transformed) are 5% and 15%, respectively. The dice score for detected lesions is 0.68. The computation time for generating delineations and reports from a WBMRI scan is within 80 s.

**Conclusions:**

Our automated tool can generate fast and accurate delineations of focal bone lesions for measuring WBMRI biomarkers, potentially assisting clinicians in staging patients with multiple myeloma and leading to personalised treatments.

### O7 The "Hungry Judge" Effect on Prostate MRI Reporting: Chronobiological Trends from 35,004 Radiologist Interpretations

#### Anton Becker, Sungmin Woo, Doris Leithner, Angela Tong, Marius E. Mayerhoefer, H. Alberto Vargas

##### Department of Radiology, NYU Grossman School of Medicine, United States

*Cancer Imaging (2024),* **24 (1):** O7

**Objectives/ Teaching Points:**

To investigate the associations between the hour of the day and Prostate Imaging–Reporting and Data System (PI-RADS) scores assigned by radiologists in prostate MRI reports.

**Material(s) and Method(s):**

Retrospective single-centre collection of prostate MRI reports over an 8-year period. Mean PI-RADS scores assigned between 0800 and 1800 h were examined with a regression model.

**Results:**

A total of 35′004 prostate MRI interpretations by 26 radiologists were included. A significant association between the hour of day and mean PI-RADS score was identified (Beta = 0.005, p < 0.001), with malignant scores more frequently assigned later in the day.

**Conclusions:**

These findings suggest chronobiological factors may contribute to variability in radiological assessments. Though the magnitude of the effect is small, it may potentially add variability and impact diagnostic accuracy.

### O8 Using ChatGPT to change free text reports into structured reports for staging CT scans

#### Mina Ibrahim, Mark Bekhit

##### Department of Radiology, Auckland City Hospital, New Zealand

*Cancer Imaging (2024),* **24 (1):** O8

**Objectives/ Teaching Points:**

Accurate and consistent radiology reporting is crucial for effective cancer staging and treatment planning. While structured reports are preferred for their clarity, existing software often requires distracting manual input, leading to the continued use of variable free-text reports. Large Language Models like ChatGPT offer a promising solution to automate the conversion of free-text CT scan reports into structured formats, enhancing communication without increasing radiologists' effort. Studies have explored ChatGPT's applications in radiology, such as simplifying reports for patients, extracting data, and generating diagnostic suggestions. Recently, promising results have been seen in converting free-text reports into structured formats for thyroid ultrasounds. However, no research has yet investigated its use in converting free-text reports for oncology staging CT scans. This study aims to assess ChatGPT's potential to standardize oncology reporting, enhance data interoperability, and improve cancer care quality.

**Material(s) and Method(s):**

We conducted a retrospective study of 101 staging CT Chest/Abdomen/Pelvis scans from our institution. CT studies were excluded if they had been reported using the preferred oncology structured template. Using GPT-4, ChatGPT (Mar 23 Version) converted original free-text reports into structured reports based on our preferred oncology template. The accuracy of these AI-generated reports was evaluated, with errors categorized as major or minor based on whether they pertained to key findings in the report conclusion.

**Results:**

Of the 101 staging CT scans, 99 (98%) were correctly structured. 15 (15%) contained major errors and 54 (53%) contained minor errors. Major errors frequently involved the omission or misplacement of breast and pelvic pathologies in the generated reports.

**Conclusions:**

ChatGPT allows radiologists to continue using free-text narrative style reports while simultaneously generating structured reports for clearer communication with clinicians and patients. Although these structured reports require oversight and minor edits to ensure accuracy, ChatGPT has the potential to streamline structured reporting workflows.

Implementing improved templates to cover breast and pelvic pathologies could further enhance the precision and completeness of these reports.

### O9 Meta-analysis of local recurrence rates following cryoablation without surgical excision for small early-stage breast cancers

#### Eelin Tan, Mok Wing Yan, Chong Jingli

##### Diagnostic Radiology, Tan Tock Seng Hospital, Singapore

*Cancer Imaging (2024),* **24 (1):** O9

**Objectives/ Teaching Points:**

Patients with early-stage breast cancer have favourable prognosis and are potential candidates for treatment de-escalation. Cryoablation shows promise as a minimally invasive alternative to surgery in patients with favourable tumour biologics. This meta analysis evaluates the efficacy of cryoablation for patients with small early-stage breast cancers.

**Material(s) and Method(s):**

A systematic search was conducted on Embase, PubMed and Google Scholar from inception to 16 June 2024. Prospective and retrospective studies of patients treated with cryoablation without surgical excision for small (≤ 15 mm) early-stage breast cancers were included. Pooled local recurrence rates were estimated with a random-effects model. Heterogeneity was assessed using tau2 and I2 statistics. Outlier detection and Egger’s test for publication bias were also performed.

**Results:**

Six studies (4 prospective, 2 retrospective) comprising 503 female patients met inclusion criteria. A total of 504 breast tumours were treated (471 invasive ductal carcinomas, 32 ductal carcinomas in-situ, 1 mucinous carcinoma). All cryoablation procedures were performed under ultrasound guidance. Clinical and imaging follow-up protocols, and adjuvant therapies varied between studies. Follow-up period after cryoablation ranged from < 6 months to 104 months. Pooled overall local recurrence rate was 1.8% (95% CI: 0.86- 3.54%). Between-study heterogeneity variance was estimated at tau2 = 0.0 (95% CI: 0.00-1.49) with an I2 value of 0% (95% CI: 0.0-74.6%), suggesting heterogeneity might not be important. No outlier studies were identified. Egger’s test suggested absence of publication bias (intercept: -0.10, 95% CI: -1.49-1.28, p = 0.89).

**Conclusions:**

This meta-analysis demonstrates that cryoablation is able to achieve excellent local tumour control rates in carefully selected patients with small early-stage breast cancers.

### O10 Misclassification analysis of a multimodal image-based deep-learning model for predicting post-operative HCC recurrence: Implications for model improvement

#### Wan Hang Keith Chiu^1^, Rex Wan-Hin Hui^2^, I-Cheng Lee^3^, Chen-Lu Wang^4^, Ho-Ming Cheng^2^, Jianliang Lu^2^, Lung-Yi Mak^2^, Tan-To Cheung^5^, Yi-Hsiang Huang^3^, Man-Fung Yuen^2^, Philip Leung-Ho Yu^4^, Wai-Kay Seto^2^

##### ^1^Department of Radiology and Imaging, Queen Elizabeth Hospital, Hong Kong; ^2^Department of Medicine, The University of Hong Kong, Hong Kong; ^3^Department of Medicine, Taipei Veterans General Hospital, Taiwan; ^4^Department of Mathematics and Information Technology, The Education University of Hong Kong, Hong Kong; ^5^Department of Surgery, The University of Hong Kong, Hong Kong

*Cancer Imaging (2024),* **24 (1):** O10

**Objectives/ Teaching Points:**

Current image-based deep-learning (DL) models for hepatocellular carcinoma (HCC) prognostication lack explainability, limiting clinical adoption. We performed misclassification analysis on our DL model to identify factors influencing its performance.

**Material(s) and Method(s):**

Recurr-NET, a multimodal DL model, integrates pre-treatment computed tomography (CT) and clinical parameters to stratify patients into high or low risk for HCC recurrence post-curative surgery. In an external cohort, it achieved AUROCs of 0.781 and 0.760 for predicting HCC recurrence at 2 and 5 years, respectively. We reviewed misclassified cases, defining true positives as high-risk patients who recurred, true negatives as low-risk patients who did not recur, false positives as high-risk patients who did not recur, and false negatives as low-risk patients who recurred. We identified parameters associated with misclassification through univariate and multivariate analyses.

**Results:**

A total of 560 patients with resected HCC (mean age 62.1 ± 12.2 years, 80.5% male, mean MELD score 7.9 ± 2.3) were included. There were 140 (25.0%) misclassifications for predicting 2-year recurrence, with 75 (13.4%) false positives and 65 (11.6%) false negatives. Ascites on CT (OR 4.316, 95% CI 1.502-12.405, p = 0.007) and MELD score (OR 1.097, 95% CI 1.002-1.201, p = 0.045) were associated with misclassification in both univariate and multivariate analyses. For predicting 5-year recurrence, there were 163 (29.1%) misclassifications, with 27 (4.8%) false positives and 136 (24.3%) false negatives. BCLC staging (OR 2.042 for BCLC-0 vs. higher stages, 95% CI 1.048-3.976, p = 0.036) and histological microvascular invasion (OR 0.615, 95% CI 0.403-0.939, p = 0.024) were associated with misclassification in univariate analysis, while ascites on CT remained the only significant factor in multivariate analysis (OR 3.690, 95% CI 1.208-11.271, p = 0.022).

**Conclusions:**

Both clinical and radiological features can impact DL model predictions. Further research on algorithm enhancements is essential to improve explainability and develop a more generalizable model for HCC prognostication post-resection.

### O11 Alkaline Phosphatase as a serum tumour marker in triaging Bone scan: our experience in Hong Kong

#### Wing Hang Luk^1^, Xin Yau Tsang^2^, Charmaine Wong^1^, Christine Tang^1^

##### ^1^Radiology, Princess Margaret Hospital, Hong Kong; ^2^The University of Hong Kong, Hong Kong

*Cancer Imaging (2024),* **24 (1):** O11

**Objectives/ Teaching Points:**

Clinicians request bone scans (BS) as a means of investigating isolated increases in serum alkaline phosphatase (ALP) levels. However, the results of these bone scans are frequently unremarkable, especially in patients with only a modest degree of ALP elevation. The value of routinely performing bone scans in this context is therefore unclear. A 3-year retrospective review was conducted to assess the correlation between raised ALP levels and the presence of occult metastatic findings on BS. The goal was to suggest a reference interval that could be used to triage BS in Hong Kong hospitals.

**Material(s) and Method(s):**

102 patients with isolated ALP increases, who were imaged with whole-body Tc-99 m HDP bone scans were reviewed. 36 patients with incomplete data or existing diseases such as renal osteodystrophy, diagnosed cancer and Paget's diseases were excluded. Patterns in liver and renal enzymes profiles, characteristic scintigraphic patterns and consultation notes were follow up and reviewed for up to 6 months after BS date.

**Results:**

51 of 66 (77%) patients had no significant bone pathology other than age-related degenerative changes or minor fractures. 11 patients had occult bone metastasis, 9 were subsequently diagnosed with underlying prostate cancer and 2 with lung cancer. 4 Paget’s disease was identified. Typically, ALP values with slight increases were subsequently self-resolved, whereas those with ALP exceeding 1.5 times of reference ranges had occult pathology. A Fisher exact test was conducted for the degree of ALP increase and its correlation with metastasis. There was a significant association between a > 1.5 times increase and metastasis, with a P value of 0.029.

**Conclusions:**

It is suggested that bone scan referrals should be prioritized if there is an isolated ALP increase over 1.5 times of the reference range.

### O12 Correlation of CT PAUSE score with MDT decision making and outcomes of cytoreductive surgery in advanced epithelial ovarian cancer

#### Anuradha Chandramohan^1^, Angelin Grace Jeslin^1^, Betty Simon^1^, Vinotha Thomas^2^, Anitha Thomas^2^, Kripa Varghese^3^, Divya Bala Thumaty^4^, Reka Kuppusamy^5^

##### ^1^Department of Radiology, Christian Medical College Vellore, India; ^2^Department of Gynecological Oncology, Christian Medical College Vellore, India; ^3^Department of Pathology, Christian Medical College Vellore, India; ^4^Department of Medical Oncology, Christian Medical College Vellore, India; ^5^Department of Biostatistics, Christian Medical College Vellore, India

*Cancer Imaging (2024),* **24 (1):** O12

**Objectives/ Teaching Points:**

To study the impact of using ‘PAUSE’ to communicate imaging findings in advanced primary epithelial ovarian cancer (EOC) on MDT decision-making and cytoreductive surgery outcomes.

**Material(s) and Method(s):**

This is a prospective study on stage III and above EOC patients who underwent CECT for disease evaluation and received advice from the Gyne-oncology MDT between July 2022 and May 2024. Two radiologists document imaging findings using PAUSE system, and correlated these with MDT decisions, treatment, and the outcome of cytoreductive surgery.

**Results:**

We studied 175 CT of 124 women with a mean age of 50.6 (SD 11.3) years and CA-125 of 1883.7 (SD 3428) IU/ml. 77 was staging CT, and rest were restaging CT following NAC. Treatment given was primary cytoreduction (n = 22), interval cytoreduction (n = 62), palliative care (n = 29), rest (n = 11) were lost to follow-up. The mean rPCI and PAUSE scores were 12 (SD 11) and 3 (SD 3), respectively. There was a significant difference in the mean rPCI among those who received MDT advice of surgery (12 (SD 7)) Vs. NAC (22 (SD 8)), and similarly the mean PAUSE score (2 (SD2) for surgery Vs. 5 (SD 3) for NAC), p < 0.001. rPCI of > 13 (OR = 11.2), ascites (OR = 6.9), unfavourable sites (OR = 8.5), and small bowel and mesenteric disease (OR = 14.1) significantly influenced the MDT advice for NAC, p < 0.001. MDT decision was not influenced by extraperitoneal disease. MDT advice was 100% concordant with treatment given. The rate of complete cytoreduction was 89.3% and only n = 2 had incomplete cytoreduction. There was only a trend toward a significant association between the outcome of cytoreductive surgery, rPCI (p = 0.07) and PAUSE score (p = 0.08).

**Conclusions:**

Components of "PAUSE" and PAUSE score significantly influenced MDT decision-making and the treatment given to patients with advanced epithelial ovarian cancer.

### O13 Choroid Plexus Uptake on Amyloid PET as an Early Predictor of Cortical Amyloid Deposition in Amyloid-negative Individuals across the Alzheimer's Disease Continuum

#### Yoon Seong Choi, Pek-Lan Khong

##### Diagnostic Radiology, National University of Singapore, Singapore

*Cancer Imaging (2024),* **24 (1):** O13

**Objectives/ Teaching Points:**

Alzheimer’s disease (AD) is marked by the progressive accumulation of amyloid plaques, with early detection being crucial for intervention strategies. The aim was to investigate the predictive capacity of choroid plexus (CP) uptake on amyloid PET for cortical amyloid deposition and future amyloid-positive conversion among amyloid-negative individuals, potentially serving as an early biomarker for AD.

**Material(s) and Method(s):**

We analyzed amyloid-negative participants from the Alzheimer's Disease Neuroimaging Initiative (ADNI, n = 634) and Harvard Aging Brain Study (HABS, n = 205) cohorts. The study analyzed amyloid PET standardized uptake value ratio (SUVR) in CP and cortical regions and cerebrospinal fluid (CSF) amyloid-beta 42 (Aβ42) levels. The prognostic value of baseline CP SUVR was assessed using linear regression for predicting the rate of future increase in cortical SUVR that was extracted by linear mixed model from repeated amyloid PET scans, and using Cox regression for predicting future amyloid-positive conversion. Baseline cortical SUVR, age, sex, education, APOE4 status were used as covariates. Mediation analysis was performed with CP SUVR as a mediator between CSF Aβ42 and cortical SUVR.

**Results:**

88 (18.5%) ADNI and 21 (13.0%) HABS participants progressed to amyloid-positive status over a median follow-up of 4.0 and 2.9 years, respectively. CP SUVR was independently prognostic for predicting the rate of cortical SUVR increase in both ADNI (b = -0.015, SE = 0.015) and HABS (b = -0.016, SE = 0.004, P < 0.001) cohorts. CP SUVR was also independently prognostic for predicting future amyloid-positive conversion in the ADNI (HR 0.06 [0.01-0.25], P < 0.001 in the ADNI) and was borderline significant in the HABS (HR 0.03 [< 0.01—1.61], P = 0.082). In mediation analysis, CP SUVR significantly mediated the association between CSF Aβ42 and cortical SUVR (b = 0.04 [0.01-0.07], P < 0.001 for indirect effect).

**Conclusions:**

CP SUVR from amyloid PET scans is a useful biomarker for predicting future amyloid deposition. It has the potential to improve the cost-effectiveness of amyloid PET scans in monitoring amyloid-negative individuals for early detection of amyloid-positive conversion. Further validation of CP SUVR is needed to evaluate its robustness and generalizability in preclinical Alzheimer's disease diagnosis.

### O14 Utility of Gallium-68 DOTATATE imaging in adrenal tumours: An Australian Experience

#### Dominic Ku^1^, Natalie Rutherford^2^

##### ^1^Radiology, John Hunter Hospital, Newcastle, Australia, Australia; ^2^Nuclear Medicine, John Hunter Hospital, Newcastle, Australia

*Cancer Imaging (2024),* **24 (1):** O14

**Objectives/ Teaching Points:**

To determine the utility of Gallium-68 DOTATATE imaging in clinical management for phaeochromocytomas.

**Material(s) and Method(s):**

Retrospective review of multidisciplinary meeting (MDM) documentation and clinical records between Jan 2022-March 2024 was conducted to assess the utility of Gallium-68 DOTATATE PET imaging in clinical decision making of adrenal tumours. Qualitative and quantitative analysis Gallium-68 DOTATATE PET and FDG PET findings including SUVmax and contralateral SUVmax, contrast CT findings including size of tumour, stage of tumour, serological markers, histopathology result and management plan was conducted.

**Results:**

A total of 150 patients were discussed in the multidisciplinary setting for phaeochromocytomas. There were 20 malignant adrenal tumours and 15 phaeochromocytomas on histopathology result. MDM decision making was analysed to determine whether or not the use of DOTATATE imaging affected management options. There was no incomplete excision of tumour and no mortality in this series.

**Conclusions:**

The use of DOTATATE imaging may confirm diagnosis of phaeochromocytoma, however given clinical suspicion and serological markers provided by the clinician, this information did not appear to alter the course of clinical management. DOTATATE low avidity and FDG PET increased avidity may predict histopathological malignant phaeochromocytoma.

### O15 Effect of furosemide on the bladder quantitative parameters in patients undergoing Gallium-68 [68 Ga]Ga-PSMA-11 PET/CT for diagnosis of prostate cancer

#### James Buteau^1^, Daniel Valenzuela^1^, Marta Tormo-Ratera^2^, Nathan Papa^1^, Renu Eapen^3^, Louise Emmett^4^, Daniel Moon^3^, Declan Murphy^3^, Michael S Hofman^1^

##### ^1^Prostate Cancer Theranostics and Imaging Centre of Excellence (ProsTIC), Molecular Imaging and Therapeutic Nuclear Medicine, Cancer Imaging, Peter MacCallum Cancer Centre, Australia; ^2^Department of Nuclear Medicine, Hospital Clínic, Spain; ^3^Division of Cancer Surgery, Peter MacCallum Cancer Centre, Australia; ^4^Department of Theranostics and Nuclear Medicine, St Vincent's Hospital Sydney, Australia

*Cancer Imaging (2024),* **24 (1):** O15

**Objectives/ Teaching Points:**

PRIMARY2 (NCT05154162) is investigating [68 Ga]Ga-PSMA-11 PET/CT for prostate cancer diagnosis. Sites of intraprostatic PSMA uptake may be masked by intense bladder activity. We aim to investigate the effects of furosemide on quantitative parameters in the bladder in patients undergoing [68 Ga]Ga-PSMA-11 PET/CT in the diagnostic setting.

**Material(s) and Method(s):**

Patients with suspected prostate cancer, PI-RADS 2 or 3 on mpMRI, and who had a pelvic only [68 Ga]Ga-PSMA-11 PET/CT were included. The non-furosemide group (PRIMARY trial) was compared with the furosemide group (PRIMARY2 trial) who received furosemide 20 mg IV at time of radiotracer injection. A pelvic-only PET/CT was acquired between 60 and 70 min, without intravenous contrast. Ordered subset expectation maximisation (OSEM) algorithm was used for tomographic reconstruction. Quantitative parameters of the bladder (SUVmax and volume) were measured by two experienced nuclear medicine physicians using an edge-detection tool. 95% confidence intervals were calculated using t-distributions.

**Results:**

Between August 30th 2019 and March 31st 2024, 95 patients were eligible with 53 in the furosemide group (F +) and 42 in the non-furosemide group (F-). Baseline characteristics were similar (F + vs F-) with median (IQR) age 63 (59–66) vs 64 (58–68) years, PSA 5.3 (4.5–7.2) vs 5.4 (4.3–7.0) ng/mL, and 38/53 (72%) vs 28/42 (67%) had PI-RADS 2 on mpMRI. Technical parameters were similar between groups, with 152 (138–182) MBq vs 152 (106–181) MBq injected [68 Ga]Ga-PSMA-11 and uptake time of 61 (60–64) min vs 63 (60–65) min. The median (IQR) SUVmax in the bladder (F + vs F-) was 11.1 (7.5–14.6) vs 34.8 (18.0–64.2), with a mean difference of 30.9 (95%CI 22.0–39.8). The bladder’s volume also considerably differed between groups with 213 (156–303) mL vs 85 (49–148) mL, and a mean difference of 126 mL (95%CI 88–164 mL).

**Conclusions:** Furosemide substantially decreases bladder uptake in patients undergoing [68 Ga]Ga- PSMA-11 PET/CT for prostate cancer diagnosis.

### O16 Naive Bayes analysis of quantitative magnetic resonance fingerprinting for brain tumour classification

#### Minh-Son To^1^, Emily Squires^2^, Anthony Tew^3^, Stephanie Withey^4^, Victoria Tan^5^, Jake Nowicky^5^, Khimseng Tew^1^, Cherie Raven^1^, Ganessan Kichenadasse^6^, Santosh Poonnoose^5^, Mark Jenkinson^7^, Marc Agzarian^1^

##### ^1^South Australia Medical Imaging, SA Health, Australia; ^2^College of Medicine and Public Health, Flinders University, Australia; ^3^Faculty of Health and Medical Sciences, University of Adelaide, Australia; ^4^Siemens Healthcare Pty Ltd, Australia; ^5^Department of Neurosurgery, Flinders Medical Centre, Australia; ^6^Department of Medical Oncology, Flinders Medical Centre, Australia; ^7^Nuffield Department of Clinical Neurosciences, University of Oxford, United Kingdom

*Cancer Imaging (2024),* **24 (1):** 016

**Objectives/ Teaching Points:**

High grade gliomas (HGGs) and brain metastases can be difficult to distinguish on conventional MRI. Magnetic resonance fingerprinting (MRF) is a novel MR technique that utilises variable spin echo parameters during a single acquisition to obtain quantitative T1 and T2 relaxation time data. The aim of this study was to assess the ability of MRF to differentiate between HGGs and metastases on a prospectively collected dataset from a single tertiary hospital in South Australia.

**Material(s) and Method(s):**

MRF acquisitions was obtained on a Siemens 3 T Magnetom Vida in 25 patients with intraaxial brain tumours: 14 high grade gliomas and 11 metastases. MRF T1 and T2 maps were segmented into enhancing solid tumour (EST), non-enhancing solid tumour (NEST), peritumoural oedema (PTO), distant oedema (DO) and cystic regions. MRF T1 and T2 relaxation times were subsequently extracted. For each region, mean, standard deviation, skewness and kurtosis were compared between tumour types using the Wilcoxon rank sum test. P values for multiple comparisons were corrected with Bonferroni correction. A Naïve Bayes classifier was used to predict tumour type based on T1/T2 relaxometry times.

**Results:**

EST T1 mean, PTO T2 standard deviation, PTO kurtosis and DO kurtosis could EST T1 mean, PTO T2 standard deviation, PTO kurtosis and DO kurtosis could differentiate HGGs and metastases (p < 0.05) before Bonferroni correction. No significant differences were found after Bonferroni correction (p < 0.00125). The Naive Bayes classifier sorted glioblastomas and metastases with an AUC of 0.79.

**Conclusions:**

MRF may differentiate HGGs and metastases based on T1 and T2 relaxation times. This has the potential to improve the pre-operative diagnosis of these tumours. Future studies may benefit from larger sample sizes, and standardised methods for image registration and region segmentation.

### 017 Contrast media extravasation in intravenous contrast-enhanced computed tomography with power injector: Incidence, follow-up outcomes, and risk factors in a cohort of 201,526 patients

#### Mei Choo Chong^1^, Ngui Yee Ping^1^, Raja Rafie Bin Raja Mahmod^2^, Liew Hui Xuan^2^, Valerie, Mon Hnin Tun^3^, Rowena Mito Maniquis^1^

##### ^1^Radiography, Changi General Hospital, Singapore; ^2^Singapore Institute of Technology, Singapore; ^3^Health Service Research, Changi General Hospital, Singapore

*Cancer Imaging (2024),* **24 (1):** 017 

**Objectives/ Teaching Points:**

This study aims to evaluate the incidence, follow-up outcomes, and risk factors associated with intravenous contrast media extravasation during CT scans utilizing a power injector in a large cohort. This research seeks to enhance our understanding of this complication and inform strategies for its prevention and management in clinical practice.

**Material(s) and Method(s):**

This retrospective study encompassed patients who received intravenous (IV) contrast-enhanced CT scans from January 2015 to December 2023. Each incidence of extravasation was digitally documented in Radiology Information System (RIS). Institutional Review Board exemption was obtained. Data on the frequency of extravasation were collected, along with patient demographics, injection parameters, and follow-up outcomes. Potential risk factors such as age, patient setting, type of examination, venous access site, injection rate, and contrast extravasated volume were analyzed using logistic regression models to identify significant predictors of extravasation.

**Results:**

577 patients encountered contrast extravasation, resulting in a rate of 0.3%. The predominant trend in follow-up outcomes was an improvement, a favourable outcome.

Notable risk factors, including age > 70 years old, female, inpatient setting, and higher injection rate demonstrated significant associations with extravasation.

**Conclusions:**

Contrast extravasation during CT scans with power injectors records higher frequency though the majority outcome is improved over the next few days. Identifying high-risk patients through predictive factors can aid in implementing preventive strategies to minimize the incidence and improve patient safety.

## Poster presentations

### P1 Navigating T Staging of Nasopharyngeal Cancer (American Joint Committee on Cancer (AJCC) 8th edition) with Case Illustrations

#### Josefina Marie Medina

##### Radiology, St. Luke's Medical Center, Philippines

*Cancer Imaging (2024),* **24 (1):** P1

**Objectives/ Teaching Points:**

Nasopharyngeal carcinoma (NPC) staging is crucial for determining prognosis and treatment. The American Joint Committee on Cancer (AJCC) 8th edition T staging provides a detailed framework for understanding the local spread of NPC. This exhibit reviews T1 to T4 stages, emphasizing key anatomic landmarks and common patterns of tumour spread that radiologists should know, illustrated with clinical cases.

**T1 Stage:**

- Tumour confined to the nasopharynx or extends to the oropharynx and/or nasal cavity without parapharyngeal extension.
- Rosenmuller fossa is the most common site for NPC to arise.
- Spread to the nasal cavity often limited to the posterior aspect.
- Inferior extension to the oropharynx is uncommon.

**T2 Stage:**

- Tumour spread to the parapharynx, involving the levator palatini muscle, pharyngobasilar fascia, and parapharyngeal fat space.
- Tumour spread can compress the eustachian tube, leading to middle ear and mastoid effusion.
- Involvement of adjacent soft tissues, including medial and lateral pterygoid muscles, and prevertebral muscles.
- Further spread may involve the carotid space, encasing the carotid artery.

**T3 Stage:**

- Tumour invasion of the skull base occurs in approximately 65% of patients.
- Common sites include the clivus, pterygoid processes, petrous apices, and sphenoid body.
- Involvement of skull base foramina (rotundum, ovale, lacerum) and canals (vidian, pterygopalatine, hypoglossal).
- Spread to the pterygopalatine fossa, leading to invasion of multiple adjacent structures.
- Tumour spread to cervical vertebra and paranasal sinuses, particularly the sphenoid and ethmoid sinuses.

**T4 Stage:**

- Intracranial invasion commonly involves the cavernous sinus and dura of the cranial fossae, leading to multiple cranial nerve palsies.
- Tumour may spread from the orbital fissures or skull base foramina.
- Extensive soft tissue involvement beyond the lateral pterygoid muscle, including the infratemporal fossa.
- Invasion of hypopharynx, parotid gland, and orbit indicates advanced local disease.

### P2 Desmoid-type Fibromatosis from Neck to Toes: A comprehensive showcase

#### Marta Braschi Amirfarzan^1^, Jordan Lemme^1^, Jyothi Jagannathan^2^

##### ^1^Radiology, Lahey Health Medical Center, United States; ^2^Imaging, Mass General Brigham, United States

*Cancer Imaging (2024),* 24 (1): P2

**Objectives/ Teaching Points:**

Desmoid-type Fibromatosis (DF) is a mesenchymal neoplasm which can involve any body part. It shows unpredictable behaviour ranging from spontaneous regression to locally aggressive disease with high morbidity and mortality. It has significant potential for recurrence.

In view of its variable biological and clinical course, the management of DF is challenging and continuously evolving. Watching and waiting is even considered a possible option in a subset of patients. In this poster, we will illustrate DF of different body parts with emphasis on their characteristics and treatment options, including the following: Pregnancy associated abdominal wall DF responding to hormonal agents, FAP associated intrabdominal DF, Sporadic multifocal extremity DF resistant to treatment, Neck DF with local bony invasion, Response of DF to radiation therapy, Response of DF to local IR therapy, Complications of intrabdominal DF from locally aggressive behaviour (small bowel obstruction, hydrosalpinx).

In conclusion, DF is a locally aggressive neoplasm of mesenchymal origin, with variable behaviour and a wide range of treatment options, including observation, cytotoxic and cytostatic therapies, surgery, radiation therapy and interventional radiology procedures.

### P3 Role of imaging in the staging and management of Carcinoma Endometrium following the 2023 update of the FIGO staging system

#### Anuradha Chandramohan, Sibangi Pramanik, Sonia Thanikaivelu, Betty Simon, Anu Eapen

##### Department of Radiology, Christian Medical College Vellore, India

*Cancer Imaging (2024),* **24 (1):** P3

**Objectives/ Teaching Points:**

Background

The International Federation of Gynaecology and Obstetrics (FIGO) staging system for endometrial cancer was updated in 2023. The update incorporates pathology and molecular classifications into the staging system and appears complex. Staging would now require a multi-disciplinary approach. There is likely to be a change in the role of imaging in the management of endometrial cancer and a shift in the referral pattern to imaging services.

Teaching points

1. In this educational exhibit we list the differences between the updated FIGO (2023) staging system and the previous 2007 staging system for Carcinoma Endometrium.
2. We display the various clinical scenarios with which patients may be referred to Radiology services and how to choose appropriate imaging modalities.
3. We display specific changes we need to make to the Endometrial Cancer reporting template to incorporate the recommendations of updated FIGO staging system.
4. We display imaging examples to demonstrate these specific points.

### P4 Adrenal Lesions—Not always an adrenal adenoma

#### James Fish, Katherine Ordidge, Anju Sahdev

##### Radiology, Barts Health NHS Trust, United Kingdom

*Cancer Imaging (2024),* **24 (1):** P4

**Objectives/ Teaching Points:**

- Adrenal incidentalomas are commonly found in routine practice on up to 5% of all abdominal CT imaging.
- Clear guidelines exist as to the management of adrenal incidentalomas; including their initial characterisation on imaging (CT, MRI and PET-CT).
- Whilst the majority (> 90%) of incidentally detected, asymptomatic adrenal lesions are benign adrenocortical adenomas, there are important differentials of which the radiologist must be aware.
- Confident imaging characterisation of adrenal lesions helps ensure that patients follow the correct clinical management pathways and avoid expensive and unnecessary follow-up, including adrenal biopsy.

**Table of Contents/Outline:**

Case based educational exhibit with multiple examples of both benign and malignant adrenal conditions and their associations with referenced teaching points to include:

**Benign Adrenal lesions:**

- Evaluation of classical imaging characteristics of adrenal adenomas on CT and MRI imaging including lipid-rich and lipid-poor subtypes with cross-correlation to functional status.
- Wider range of benign adrenal pathology distinct from adrenal adenomas, including paragangliomas, adrenal rests, myelolipomas, angiomyolipomas, congenital adrenal hyperplasia (CAH), adrenal cysts and ganglioneuromas.

**Malignant Adrenal lesions:**

- Delineate imaging characteristics which should raise the suspicion of adrenal malignancy; both primary and secondary malignancy and in turn avoid unnecessary investigations or upstaging of patients.
- Array of malignant adrenal pathology including phaeochromocytomas, adrenocortical carcinomas, metastases to the adrenal gland, malignant neuroendocrine tumours and composite tumours.

**Indeterminate Adrenal lesions:**

- Lesions that are not clearly benign or malignant on initial imaging remain a challenge to radiologists. Correlation with biochemical status, clinical assessment and further imaging modalities including nuclear medicine investigations can help confirm a diagnosis.<div class="NodiCopyInline">Lesions that are not clearly benign or malignant on initial imaging remain a challenge to radiologists. Correlation with biochemical status, clinical assessment and further imaging modalities including nuclear medicine investigations can help confirm a diagnosis.</div>

### P5 Revised FIGO 2023 Staging of Endometrial Cancer: What Radiologists Should Know

#### Jia Huey Wong^1^, Aslam Sohaib^2^

##### ^1^Radiology, IKN, Malaysia; ^2^Radiology, Royal Marsden Hospital, United Kingdom

*Cancer Imaging (2024),* **24 (1):** P5

**Objectives/ Teaching Points:**

There have been significant advancements in the understanding of the pathology, molecular characteristics, treatment options and clinical outcomes of endometrial carcinoma. This has necessitated FIGO to revise its staging classification to incorporate and reflect these new knowledge and data.

The key changes in the current staging system will be highlighted along with comparison to the previous FIGO 2009 system. Each stage of endometrial carcinoma will be described in detail and illustrated by MRI examples. The impact it has upon the MRI interpretation, radiological staging and subsequent management of patients will be discussed.

Therefore, it is essential for radiologists to acquaint themselves with the updated staging classifications and comprehend its influence on the management of endometrial carcinoma.

### P6 Treatment Response Criteria in Precision Prostate Oncology: Overview of PCGW3- modified RECIST 1.1 Assessment

#### Jiyae Lee^1^, John Malinowski^2^, Hyesun Park^3^, Elisa Franquet-Elia^4^, Marta Braschi-Amirfarzan^1^, Lacey McIntosh^5^

##### ^1^Diagnostic Radiology, Lahey Hospital & Medical Center, United States; ^2^Diagnostic Radiology, UMass Memorial Medical Center, United States; ^3^Nuclear Medicine and Diagnostic Radiology, Lahey Hospital & Medical Center, United States; ^4^Nuclear Medicine, UMass Memorial Medical Center, United States; ^5^Nuclear Medicine and Diagnostic Radiology, UMass Memorial Medical Center, United States

*Cancer Imaging (2024),* **24 (1):** P6

**Objectives/ Teaching Points:**

Recent advancements in PSMA-directed diagnosis and treatment of prostate cancer have led to an explosion in research and new treatment regimens. As with other novel therapies, these new treatments may demonstrate atypical imaging findings, especially early in treatment, which require specific and modified response assessment criteria. At present, many clinical trials for patients undergoing novel prostate cancer treatment utilize an integrated Prostate Cancer Working Group 3 (PCWG3)-modified Response Evaluation Criteria in Solid Tumors (RECIST) 1.1 criteria.

The goals of this poster are to review response assessment criteria for prostate cancer lesions using PCWG3 and RECIST 1.1. The reader will review the evolution of criteria, learn how to select and document visceral and osseous disease assessment at baseline, calculate tumour burden, review methodology for tracking lesions on follow up, assigned timepoint specific response assessments, and formulate an integrated over response assessment.

This educational exhibit will use illustrative figures and example bone scan/CT to demonstrate examples of baseline and various response scenarios in prostate cancer patients undergoing treatment and instruct the reader how to formulate an accurate assessment. Specific teaching points include baseline lesion selection, treatment flare progression at first follow up (2 + 2 rule), and progression after first follow up.

Concluding, in the era of precision prostate cancer treatment, radiologists are required to have increased awareness and familiarity with novel response assessment criteria. As important members on the cancer care team, radiologists must be aware and familiar with PCWG3 modified RECIST 1.1 response assessment criteria, both in the setting of clinical trial reading and routinely at the workstation.

### P7 Every aortic valve tells a story – a pictorial essay of CT cardiac gated imaging of the aortic valve

#### Wai Keat Lau^1^, Ching Ching Ong^2^, Yun Song Choo^2^, Yinghao Lim^3^, Ivandito Kuntjoro^3^, Lynette Li San Teo^2^

##### ^1^NUHS, Singapore; ^2^Department of Diagnostic Imaging, National University Hospital, Singapore; ^3^Department of Cardiology, National University Hospital, Singapore

*Cancer Imaging (2024),* **24 (1):** P7

**Objectives/ Teaching Points:**

Aortic stenosis is the most common valvular heart disease in developed countries. Transcatheter Aortic Valve Implantation (TAVI) is now an established treatment option in patients who are ineligible for surgery or high risk surgical candidates. Over the years, TAVI is also increasingly being used in patients at intermediate risk for conventional surgical valve replacement. With advances in computed tomography (CT) imaging, CT is now the gold standard for pre-procedural evaluation of potential TAVI patients. Most patients with severe aortic stenosis have degenerate valves and these are usually seen as trileaflet calcified aortic valves. There are also less common causes of aortic valve dysfunction that undergo TAVI procedures and these include bicuspid aortic valve morphology, rheumatic heart disease and previous radiation to the chest with involvement of the aortic valve leaflets. Of note, the bicuspid aortic valve annulus has a more elliptical shape with asymmetric annular calcification; this can affect transcatheter valve frame expansion resulting in increased procedural complications such as paravalvular leak and aortic root injury. This pictorial essay demonstrates the typical degenerate trileaflet aortic valve with a high Agatston score and also aims to highlight the other less common causes of aortic valve dysfunction on cardiac gated CT heart scans. These include bicuspid aortic valves, as well as valves affected by rheumatic heart disease or prior radiation. The distinctive features of each will be discussed.

### P8 Tuberculous Arthropathy: What the Radiologist Should Know—A Review of Imaging, Microbiological and Treatment Considerations

#### Aric Lee^1^, Amanda Koh^2^

##### ^1^Department of Diagnostic Imaging, National University Hospital, Singapore; ^2^Tuberculosis Control Unit, Tan Tock Seng Hospital, Singapore

*Cancer Imaging (2024),* **24 (1):** P8

**Objectives/ Teaching Points:**

Tuberculous (TB) arthropathy remains a concern in endemic countries and among migrant populations. Early diagnosis and anti-TB therapy are crucial for functional recovery, yet clinical features can be non-specific, and concomitant pulmonary TB is sometimes absent. Radiologists should maintain a sufficient degree of suspicion in high-risk patients presenting with progressive joint symptoms.

Patients typically present with subacute-chronic monoarthritis characterised by pain, swelling and functional impairment, predominantly affecting medium to large joints. Two distinct pathologic variants exist: an indolent granular variant seen in adults, and a juvenile caseous exudative variant associated with bone erosion, abscesses and sinuses.

We present a case series demonstrating radiological findings important for diagnosis and management. Initial radiographs often reveal joint effusions and peri-articular osteopaenia, followed by cortical and joint destruction. CT is useful in complex anatomical regions and to guide biopsy. MRI allows identification of marrow oedema, necrosis, abscesses, and synovial changes (including ‘rice’ bodies), as well as sinus tracts and extra-articular features including tenosynovitis, bursitis and myositis.

Microbiological confirmation through joint aspiration or biopsy is essential, with samples undergoing acid-fast bacilli smears, culture, and nucleic acid amplification tests (NAATs). Advanced NAAT-rifampicin resistance assays like BDMax and Xpert MTB/RIF enhance sensitivity and complement traditional diagnostics by rapidly differentiating TB from nontuberculous mycobacteria and providing crucial drug susceptibility information.

Nonetheless, culture remains necessary for final diagnosis. Synovial fluid analysis, while commonly performed, often yields non-specific results.

Prompt initiation of anti-TB therapy often obviates surgery and results in favourable functional outcomes. In Singapore, drug-sensitive TB arthropathy is treated with a rifampicin-containing regimen for nine months, though the optimal treatment duration remains uncertain. Surgical interventions like debridement, arthroplasty or arthrodesis may be required for large abscesses, non-viable tissue, or failed medical management. Follow-up relies on clinical assessment due to potential discordance with inflammatory markers and radiographic indicators.

### P10 Cancer Imaging Peer Learning: Elevating cancer imaging practice together

#### Marta Braschi-Amirfarzan, Richard Thomas, Hyesun Park, Jennifer Broder

##### Radiology, BILH Lahey Health Medical Center, United States

*Cancer Imaging (2024),* **24 (1):** P9

**Objectives/ Teaching Points:**

Peer learning is a system of peer review in which colleagues provide non-judgemental feedback to each other in an effort to elevate performance across the group. Ongoing feedback about both discrepancies and great calls is given to individuals, high-yield teaching points are shared in routine conferences, and systems improvements enacted where feasible. In the modern oncology era, with a new arena of precision anticancer treatments, Cancer Imaging Peer Learning (CIPL) ensures general radiology practice meets standards of oncology care.

Application of CIPL in our department:

- CIPL assists in the choice of the optimal imaging modality: E.g. including appropriate tracer specific PET/CT and MRI vs CT for assessing response to cytostatic agents.
- CIPL elevates practice by reducing knowledge gaps about specific therapies: E.g., reviewing the atypical patterns of response to treatment and the side effects of the newest therapies.
- CIPL elevates practice by sharing diagnostic successes: E.g., detection of grade I toxicity.
- CIPL identifies systems issues to improve interpretation: E.g., comparison with baseline and nadir scans is needed for correct response assessment.
- CIPL identifies patterns of diagnostic discrepancies across radiologists: E.g., perirenal involvement from HemOnc malignancies, sarcoid like reaction versus nodal progression on immunotherapy.
- CIPL generates improvements by incorporating feedback from oncologists: E.g., a succinct impression providing an overall assessment of tumour burden in the impression instead of listing changes at individual sites of metastases.
- CIPL facilitates learning between subspeciality sections: E.g., a hip synovial sarcoma and pelvic adenopathy in the same pelvic MRI suggests secondary lymphoma.

In conclusion, CIPL ensures that the general radiologist, in an environment free of judgment, keeps abreast with the continuously evolving precision cancer treatments, to provide better care to patients with cancer.

### P10 Metabolic imaging can distinguish ovarian cancer subtypes and detects their early and variable responses to treatment

#### Ming Li Chia^1^, Kevin Brindle^2^

##### ^1^Radiology, National University Health System (NUHS), University of Cambridge, Singapore; ^2^Cancer Research UK Cambridge Institute, Department of Biochemistry, University of Cambridge, United Kingdom

*Cancer Imaging (2024),* **24 (1):** P10

**Objectives/ Teaching Points:**

High grade serous ovarian cancer(HGSOC) displays two metabolic subtypes; a high OXPHOS subtype with elevated expression of genes encoding electron transport chain components, increased oxygen consumption and increased chemosensitivity due to oxidative stress and a low OXPHOS subtype that exhibits glycolytic metabolism and higher drug resistance. This project investigated the potential of magnetic resonance spectroscopy (MRS) of hyperpolarized [1-13C]pyruvate metabolism to be used clinically to distinguish low OXPHOS and high OXPHOS tumour deposits in HGSOC patients and to detect their differential responses to treatment.

**Material(s) and Method(s):**

HGSOC cells, derived from the ascites of stage 3–4 HGSOC patients, were maintained as patient derived organoids (PDO) and implanted into mice subcutaneously. The resulting tumours were imaged with MRS (hyperpolarized [1-13C]pyruvate metabolism), MRI (Dynamic Contrast Enhanced (DCE)) and with PET (measurements o2f—deoxy-2-[fluorine-18]fluoro-D-glucose uptake). PDO 2(carboplatin sensitive) and PDO 5(Carboplatin resistant) tumour models were treated with i.v. Carboplatin (50 mg/kg) or drug vehicle weekly, with imaging at baseline and weekly thereafter.

**Results:**

In patient-derived organoids and xenografts, the low OXPHOS subtypes (PDOs 1 and 5) showed increased lactate dehydrogenase activity, increased monocarboxylate transporter 4 expression and increased lactate labelling in 13C MRS measurements of hyperpolarized [1-13C]pyruvate metabolism. Both 13C MRS measurements of hyperpolarized [1-13C]pyruvate metabolism and PET measurements of [18F]FDG uptake detected response to Carboplatin treatment in high OXPHOS xenografts(PDO 2) that were Carboplatin-sensitive and non-response in low OXPHOS xenografts(PDO 5) that were Carboplatin-resistant.

**Conclusions:**

Patient-derived xenografts representative of the different HGSOC metabolic subtypes showed differences in glycolytic metabolism that could be detected with hyperpolarized [1-13C]pyruvate. Myc and EGFR amplification are likely to be responsible for driving increased lactate labelling in the low OXPHOS metabolic subtypes. 13C MRS imaging of hyperpolarized [1-13C]pyruvate metabolism has the potential to be used clinically to distinguish low OXPHOS and high OXPHOS tumour deposits in HGSOC patients and to detect their differential responses to treatment.

### P11 Performance of radiologists and radiology residents in paediatric appendicular fracture detection with and without the help of Artificial Intelligence – a snapshot study

#### Praveen M Yogendra^1^, Goh Adriel Guang Wei^1^, Yee Sze Ying^1^, Jawan Freda^1^, Koh Kelvin Kay Nguan^1^, Tan Timothy Shao Ern^2^, Woon Tian Kai^2^, Yeap Phey Ming^1^, Tan Min On^1^

##### ^1^Radiology, Sengkang General Hospital, Singapore; ^2^Diagnostic and Interventional Imaging, KK Women’s and Children’s Hospital, Singapore

*Cancer Imaging (2024),* **24 (1):** P11

**Objectives/ Teaching Points:**

We aim to evaluate the accuracy of radiologists and radiology residents in detection of paediatric appendicular fractures with and without the help of a commercially available fracture detection Artificial Intelligence (AI) solution in the hopes of showing potential clinical benefits in a general hospital setting.

**Material(s) and Method(s):**

This was a retrospective study. 3 Associate/Junior Consultant (AC) radiologists and 3 Senior Residents/Senior Registrars (SR) in Radiology acted as readers in this study. 1 reader from each human group interpreted the radiographs with the aid of AI. Cases were categorized into concordant and discordant cases between each interpreting group.

Discordant cases were then further evaluated by 3 independent subspecialty radiology consultants to determine the final diagnosis. A total of 250 anonymised paediatric patient cases (aged 2 to 15 years) who presented to a tertiary general hospital which has a Children’s emergency were retrospectively collected. Main outcome measures include: presence of fracture, accuracy of readers with and without the help of AI, total time taken to interpret the radiographs.

**Results:**

The AI solution showed the highest accuracy (Area Under the Curve, AUC 0.97). The two readers aided with AI had higher AUCs compared to readers without AI support. The average accuracy of AC subcategory (AUC 0.84) and SR (AUC 0.88) subcategory were lower compared to the AI algorithm alone.

**Conclusions:**

To our knowledge, this is the first local study looking at performance of AI against human readers when reading paediatric appendicular fractures. Current commercially available fracture detection AI solutions show potential to improve fracture detection in paediatric limbs and pelvises. This in turn could reduce recall rates and misdiagnoses. Such a solution should be implemented in emergency centres to allow for better quality of care.

### P12 Hiding in Plain Sight: A Case-based Discussion on Cardiac Angiosarcomas and Other Rare Intravascular Tumours

#### Zongchen Li, Yun Song Choo

##### Department of Diagnostic Imaging, National University Health Systems, Singapore

*Cancer Imaging (2024),* **24 (1):** P12

**Objectives/ Teaching Points:**

Teaching Points:

1. Clinical manifestations, imaging features, radiopathological correlation and complications of cardiac angiosarcomas.
2. Other rare intravascular tumours and their imaging features, including pulmonary artery sarcomas, intravascular leiomyomatosis, and vascular leiomyosarcomas.

Background / Outline:

Tumours located within the cardiovascular system are rare, regardless if they originated there or from elsewhere. Histologically, some of these are comprised of vascular channels or blood-filled cavities while others are more solid soft tissue masses, and this influences their imaging characteristics.

Vascular tumours tend to mimic the blood pool on multiphasic imaging, a feature capitalised on to detect and characterise them in solid organs. Solid tumours, while not enhancing as much as the blood pool, can be discerned by their enhancement relative to surrounding tissues especially if they are more aggressive or hypervascular.

When these tumours are located within the blood pool itself, these characteristics may instead aid them in evading detection by blending in with the blood or by masquerading as the much more common hypoattenuating intravascular entity – a clot.

In this primary case of an elusive metastatic cardiac angiosarcoma, its appearances on multiple imaging modalities are demonstrated—including US, CT in the pre-contrast, early and late arterial, portal venous and delayed phases, PET CT and MRI. Its clinical manifestations and associated complications such as haemorrhage and Kasabach-Merritt syndrome are further discussed.

A series of secondary cases brings awareness to other rare intravascular tumours that can similarly hide in plain sight.

### P13 Whole Body Magnetic Resonance Imaging in Paediatric Oncology—A 12 year Retrospective Review of 53 Examinations Performed at a Large Tertiary Centre

#### Timothy Shao Ern Tan, Phua Hwee Tang

##### Department of Diagnostic and Interventional Imaging, KK Women's and Children's Hospital, Singapore

*Cancer Imaging (2024),* **24 (1):** P13

**Objectives/ Teaching Points:**

To review the utilisation of whole-body magnetic resonance imaging (WB-MRI) examinations performed for paediatric cancers at KK Women's and Children's Hospital, Singapore.

**Material(s) and Method(s):**

WB-MRI has emerged as a valuable imaging tool in enabling a comprehensive ‘all-organ’ approach to screening, staging and monitoring disease extent in many cancers, providing essential anatomical and functional information. It is a rapid and sensitive method, particularly in paediatrics, mitigating ionizing radiation and providing excellent soft tissue depiction.

Our local institution WB-MRI protocol consists mainly of three sequences (short tau inversion recovery, T1-weighted fast spin echo, diffusion weighted imaging), performed on a 3 Tesla clinical MRI system. No contrast medium is routinely administered.

Clinical and imaging data of paediatric patients who underwent WBMRI examinations for oncologic indications between March 2011 and December 2023 were retrospectively reviewed.

**Results:**

A total of 31 patients (14 males, median age 15 years, range 4.8–26 years, scan duration ranging between 45–90 min) underwent 53 WB-MRI examinations for oncologic indications. These indications comprised of cancer predisposition syndromes (18/53 for 8 patients), neurofibromatosis (2/53), lymphoma (11/53), Langerhans cell histiocytosis (1/53), and other solid-organ neoplasms (21/53). Forty-three examinations (28 patients) detected suspicious lesions, mainly: local tumour recurrence (4/53), nodal (13/53), bone (16/53), and solid organ lesions (16/53, including 7/53 lung nodules > 9 mm). Of these, 11(26%) were screening examinations for patients with cancer predisposition syndromes.

**Conclusions:**

WB-MRI is useful for cancer screening in at-risk patients, especially in older children, as well as in detecting lesions across multiple body sites in paediatric oncology, particularly nodal and osseous/marrow disease. Lung lesions > 9 mm in size are also detectable on WBMRI and may be a useful adjunct to CT thorax for follow up for dominant lung nodules. Local paediatric WB-MRI protocols should be further tailored towards specific conditions, optimised towards shorter scan times and complementing functional imaging studies to improve diagnostic yield.

### P14 Ultrasound techniques: Opportunities and Innovations in Head and Neck Cancer

#### Adebayo Alade^1^, George Bitar^1^, Alina Dragan^1^, Nicos Fotiadis^1^, Joshua Shur^1^, Vinidh Paleri^2^, Professor Kevin Harrington^3^, Derfel ap Dafydd^1^

##### ^1^Radiology, Royal Marsden NHS Foundation Trust, United Kingdom; ^2^Surgery, Royal Marsden NHS Foundation Trust, United Kingdom; ^3^Oncology, Royal Marsden NHS Foundation Trust, United Kingdom

*Cancer Imaging (2024),* **24 (1):** P14

**Objectives/ Teaching Points:**

Developments in cancer care, particularly within the realm of head and neck cancer present opportunities for radiologists to work in collaboration with surgeons and oncologists to positively impact patient care. These advancements include intra-oral ultrasound guided trans-oral robotic surgery; saline aided intra-oral ultrasound-guided biopsy; and ultrasound-guided intra-tumoural administration of directly injected therapies.

Intra-oral ultrasound guided trans-oral robotic surgery:

- Limitations of trans-oral robotic surgery (TORS) include the surgeon's restricted ability to palpate and assess the pharynx, combined with alterations in intra-operative patient positioning due to neck extension, mouth retraction and tongue manipulation.
- Intra-oral ultrasound-guided TORS is a real-time, collaborative approach carried out in theatre. Under general anaesthesia tumour boundaries and depth can be cognitively mapped providing real-time feedback on tumour location and local anatomy.
- Ex-vivo ultrasound assessment can also be performed to assess for macroscopic margins before the end of surgery.

Saline aided intra-oral ultrasound guided biopsy:

- Tissue sampling of retropharyngeal lesions is a technical challenge. Most are not visible (and hence not targetable) with standard percutaneous ultrasound. Biopsy with CT guidance often requires a trans-facial approach and risk injury to local structures including the carotid artery.
- Under general anaesthesia intracavitary flooding with saline acts as a coupling agent to facilitate fine needle aspiration, core biopsy, TORS excision or a combination of techniques.

Ultrasound-guided directly injected therapies (DIT):

- DIT is a developing research field comprised of a range of therapies including immunotherapy, modified oncolytic viruses and peptides, bacteria and chemotherapy.
- The majority of DIT will be performed by oncologists, but a select number of cases will be referred to radiology for image-guided injection.
- The goal of DIT is the higher concentration of drug within the target tumour, and the attenuated systemic adverse event profile.

### P15 Evaluation of treatment response in HCC after TARE

#### Jeevitesh Khoda, Saugata Sen, Dayananda Lingegowda, Sumit Mukhopadhyay, Aditi Chandra, Argha Chatterjee, Priya Ghosh, Anurima Patra, Anisha Gehani

##### Department of Radiology, Tata Medical Center, India

*Cancer Imaging (2024),* **24 (1):** P15

**Objectives/ Teaching Points:**

Learning Objectives:

1. To understand the role of Transarterial Radioembolization Embolization (TARE) in HCC.
2. To understand the post TARE imaging evaluation of HCC.
3. To sensitize readers on various imaging patterns in post TARE imaging.

TARE is a form of locoregional treatment of HCC done with yttrium 90 (Y90) using a transarterial catheter that delivers microspheres coated with Y90 to HCC. The high energy beta particles emitted by Y90 destroys the tumour locally with minimal damage to normal tissue. Following TARE, initially, arterial enhancement may be seen which is followed by pseudoprogression characterized by necrosis in the first three months. The following months shows a gradual decrease in size of the tumour. Perfusion wedge shaped defects and eventual fibrosis may show delayed enhancement and should not be confused with viable tumour.

Few weeks following TARE, rare but dangerous complications like radioembolization-induced liver disease may be seen which may present with atypical imaging features. Response assessment is usually done following the LI RADS algorithm but EASL or mRECIST criteria may also be used. Response assessment might be challenging, especially in the early months, following TARE due to the reasons elaborated above.

The goal of this educational paper is to elicit response assessment imaging, highlight pitfalls and potential complications following TARE on case based approach.

### P16 Histogram based approach of apparent diffusion coefficient in differentiating between glioma grade 2 and grade 3 tumours

#### Duy Ngo Quang^1^, Nguyen Duy Hung^2^, Quach Thuy Duong^2^, Nguyen Thi Hai Anh^3^

##### ^1^Radiology Department, Vietnamese Germany Friendship Hospital, Vietnam; ^2^Radiology Department, Ha Noi Medical University, Vietnam; ^3^Radiology Department, Alexander Lepeve Hospital, France

*Cancer Imaging (2024),* **24 (1):** P16

**Objectives/ Teaching Points:**

This retrospective study evaluates the value of histogram analysis in differentiating between glioma grade 2 and grade 3 tumours on the apparent diffusion coefficient (ADC). The whole solid tumour was assessed, excluding its cystic, calcified, haemorrhagic, and necrotic portions.

**Material(s) and Method(s):**

We retrospectively evaluated 102 glioma patients on brain contrast-enhanced magnetic resonance imaging (MRI). These patients underwent surgery with the pathological result of grade 2 and grade 3 gliomas. We calculated the histogram analysis (HA) parameters, including mean, median, maximum, minimum, kurtosis, skewness, entropy, standard deviation (SD), mean of positive pixels, and uniformity of positive pixels by whole solid tumour VOI placement. We compared these parameters using the independent sample Ttest, Mann–Whitney U test, and area under the receiver operating characteristic curve (AUC) to determine their optimal cut-off, sensitivity (Se), and specificity (Sp).

**Results:**

This study included 102 patients with 49 grade 2 gliomas and 53 grade 3 gliomas. The ADCmean, ADCmedian, and ADCmin parameters have high values in discriminating between glioma grade 2 and grade 3, with AUC, cutoff, sensitivity, and specificity are 0.947, 1329, 94.7%, 85.7% in ADCmean, 0.937, 1325, 94.7%, 81% in ADCmedian và 0.897, 671.5, 89.5%, 81% in ADCmin, respectively. ADCmax and ADC MPP are also good at differentiating between these groups with AUC > 0.5. The other varies are less critical with AUC < 0.5.

**Conclusions:**

The HA parameters are valuable in differentiating between grade 2 and 3 glioma. In measuring HA parameters, the whole solid tumour VOI placement method, excluding cystic, calcified, haemorrhagic, and necrotic portions, is significantly better than ROI (region of interest) placement.

### P17 MRI-based radiomics and circulating plasma gelsolin as biomarkers of chemoresistance in epithelial ovarian cancer

#### Emma Gerber^1^, Rahul Singh^1^, Cheuk Nam Hwang^1^, Alice Wong^2^, Dylan Burger^3^, Benjamin K. Tsang^3^, Karen K. L. Chan^4^, Elaine Y. P. Lee^1^

##### ^1^Department of Diagnostic Radiology, University of Hong Kong, China; ^2^School of Biological Sciences, University of Hong Kong, China; ^3^Chronic Disease Program, Ottawa Hospital Research Institute, Canada; ^4^Department of Obstetrics and Gynecology, University of Hong Kong, China

*Cancer Imaging (2024),* **24 (1):** P17

**Objectives/ Teaching Points:**

Resistance to platinum-based chemotherapy remains a challenge faced by epithelial ovarian cancer (EOC) patients. Currently, there are no biomarkers to predict chemoresistance. Circulating plasma gelsolin (pGSN) was previously shown to predict chemoresistance in treatment-naïve EOC. Here, we aimed to expand upon pGSN as biomarker by incorporating MRI-based radiomics to improve prediction of chemoresistance in EOC patients.

**Material(s) and Method(s):**

In this retrospective study, we used the serum collected from 40 EOC patients with paired baseline MRI from the University of Hong Kong between 2016 and 2020. Circulating pGSN was measured from the serum using sandwich ELISA. Radiomic features were extracted from the primary tumour on the apparent diffusion coefficient (ADC) map (b = 0,400,800 s/mm2) using PyRadiomics (version 3.1). Highly correlated features (Spearman correlation coefficient of > 0.9) were removed. Highly repeatable features were selected using elasticnet regression iterated over 100 times for fivefold stratified cross-validations (SCVs) and only those selected > 300 times were considered for further analysis. Mann–Whitney U-test and f-test were conducted to consider statistically significant and repeatable features (p < 0.05) for model building. Grid-search tenfold SCVs was utilized to optimize the hyper-parameters of Gaussian Naïve Bayes classifier to build the prediction model using 6 highly significant features including pGSN.

**Results:**

Among the 40 EOC patients (56 ± 11 years old), the majority presented at advanced stage (FIGO III-IV, n = 26). Thirty-three patients were chemosensitive and seven were chemoresistant (progression free interval < 12 months). The proposed framework achieved the following mean performance metrics: area under the curve (0.929), sensitivity (0.900), specificity (0.908) and accuracy (0.920) in predicting chemoresistance. Both pGSN and flatness from the original shape feature contributed more “weight” in the prediction.

**Conclusions:**

The combined model demonstrated excellent diagnostic ability in predicting chemoresistance in EOC patients, which could promote tailored therapeutics.

### P18 Deep Learning Model for Degenerative Cervical Spine MRI: Impact on Reporting Efficiency and Accuracy

#### Aric Lee^1^, Junran Wu^2^, Changshuo Liu^2^, Andrew Makmur^1^, Yonghan Ting^1^, Shannon Lee^1^, Matthew Chan^1^, Desmond Lim^1^, Vanessa Khoo^1^, Jonathan Sng^1^, Han Yang Ong^1^, Amos Tan^1^, Shuliang Ge^1^, Faimee Erwan Muhamat Nor^1^, Yi Ting Lim^1^, Joey Beh^3^, Qai Ven Yap^4^, Jiong Hao Tan^5^, Naresh Kumar^5^, Beng Chin Ooi^2^, James Thomas Patrick Decourcy Hallinan^1^

##### ^1^Department of Diagnostic Imaging, National University Hospital, Singapore; ^2^Department of Computer Science, School of Computing, National University of Singapore, Singapore; ^3^Department of Radiology, Ng Teng Fong General Hospital, Singapore; ^4^Biostatistics Unit, Yong Loo Lin School of Medicine, National University of Singapore, Singapore; ^5^University Spine Centre, University Orthopaedics, Hand and Reconstructive Microsurgery (UOHC), National University Health System, Singapore

*Cancer Imaging (2024),* **24 (1):** P18.

**Objectives/ Teaching Points:**

Cervical spine MRI is crucial in the evaluation of degenerative cervical spondylosis but time-consuming to report. We sought to assess whether a deep learning (DL) model would enhance the speed and accuracy of radiologists with varying experience in reporting such MRIs.

**Material(s) and Method(s):**

We utilized a previously developed transformer-based DL model to classify spinal canal (grades 0/1/2/3) and neural foraminal (grades 0/1/2) stenoses at each disc level using published scales. The test set included random MRIs (axial T2- weighted gradient-echo, sagittal T2- weighted images) from December 2015 to August 2018 from an external hospital, excluding cases with instrumentation. Two experienced musculoskeletal (MSK) radiologists (12-years’ experience each) labelled the test set in consensus (reference standard). Ten radiologists (1–7 years’ experience) performed stenosis gradings with and without DL model use, with a 1-month washout period between sessions. Interpretation time (seconds) and interobserver agreement (Gwet’s kappa) were assessed.

**Results:**

The test set comprised 50 cervical spine MRIs (2555 images) from 50 patients (mean age = 60 ± 14;13(26%)women). DL use significantly decreased mean interpretation time from 159–490 (SD27-649) to 90–182 s (SD57-218,p < 0.001), with the largest improvement among in-training radiologists (-308 s). DL use improved interobserver agreement across all stenosis gradings. For dichotomous spinal canal grading, in-training radiologists had the largest improvement (κ = 0.77 vs 0.63,p < 0.001), with smaller improvements for general (κ = 0.77 vs 0.65,p = 0.008) and MSK (κ = 0.67 vs 0.60, p = 0.052) radiologists.

Conversely, for dichotomous neural foraminal grading, MSK radiologists had the largest improvement (κ = 0.72 vs 0.60,p < 0.001) with smaller improvements for general (κ = 0.73 vs 0.66,p = 0.003) and in-training (κ = 0.70 vs 0.67,p = 0.230) radiologists. Notably, DL model performance alone was equivalent or superior to all readers.

**Conclusions:**

DL model use improved radiologists’ interpretation time and interobserver agreement for degenerative cervical spine MRI, regardless of experience level. However, its impact on diagnostic accuracy was more varied. While DL models hold promise, further refinement is necessary to maximise their effectiveness in clinical practice.

### P19 Change in diffusion-weighted imaging after induction therapy for the prediction of outcomes in advanced nasopharyngeal carcinoma

#### Qiyong Hemis Ai^1^, Ho-sang Leung^2^, Tsz Shing A. Kwong^2^, Yannis Yan Liang^1^, Linfang Lan^1^, Lun M. Wong^2^, Ann D. King^2^

##### ^1^Department of Health Technology and Informatics, The Hong Kong Polytechnic University, Hong Kong; ^2^Department of Imaging and Interventional Radiology, The Chinese University of Hong Kong, Hong Kong

*Cancer Imaging (2024),* **24 (1):** P19

**Objectives/ Teaching Points:**

To investigate the predictive value of diffusion weighted imaging (DWI) performed before and after induction chemotherapy (post-IC) for long-term outcome in patients with locoregionally advanced nasopharyngeal carcinoma (adNPC).

**Material(s) and Method(s):**

This study retrospectively reviewed pre- and post-IC DWIs of 63 eligible patients with adNPC who were treated with IC followed by concurrent chemoradiotherapy (CCRT). DW images with two b-values (0 and 1000 s/mm2) were used to fit a mono-exponential diffusion model to generate the conventional apparent diffusion coefficient (ADC) map. The primary tumour on the pre- and post-IC ADC maps was manually contoured. The mean values of pre- and post-IC ADC (ADCpre and ADCpost-IC), and changes in ADC between pre- and post-IC scans, which were defined as ΔADC% = [(ADCpost-IC—ADCpre)/ ADCpre *100%] were calculated and correlated with disease free survival (DFS) using Cox regression analysis. Significant parameters, together with age, sex, and T-staging, Nstaging, and overall stage were then added into multivariate Cox regression to identify the independent parameters for the prediction of outcomes.

**Results:**

The 3-year DFS was 86.3%. Univariate analysis showed that high ΔADC% (greater rise in The 3-year DFS was 86.3%. Univariate analysis showed that high ΔADC% (greater rise in ADC on post-IC scan compared with that on pre-treatment scan) correlated with good DFS (HR = 0.959, p = 0.02); but not ADCpre and ADCpost-IC (p = 0.30 and 0.10, respectively). After adjusting the confounding factors, ΔADC% remained independent of being the predictor for DFS (HR = 0.946, p < 0.01).

**Conclusions:**

Percentage change in ADC value between pre- and post-IC scans, but not absolute ADC values on the pre- and post-IC scans had the potential to be the predictor of long-term treatment response in patients with locoregionally adNPC.

### P20 Neuroimaging Biomarkers for Brain Age Prediction in a Singaporean Ethnic Chinese Population: A Deep Learning Approach

#### Bhanu Prakash KN^1^, Arvind CS^2^, Ashutosh Raj Shrestha^3^, Oliver James Nickalls^4^, Parag Ratnakar Salkade^4^, Ho Chi Long^4^

##### ^1^Bioinformatics Institute, ASTAR, Singapore; ^2^Clinical Data Analytics & Radiomics, Bioinformatics Institute, Agency for Science, Technology and Research, Singapore; ^3^Information Technology, Vellore Institute of Technology, India; ^4^Department of Diagnostic Radiology, Sengkang General Hospital; Duke-NUS Medical School: NUS Yong Loo Lin School of Medicine, Singapore

*Cancer Imaging (2024),* **24 (1):** P20

**Objectives/ Teaching Points:**

As the global population ages, the prevalence of neurodegenerative diseases is expected to rise significantly. Developing accurate and accessible neuroimaging biomarkers for brain age estimation and understanding brain aging gives a deeper insight to the complex processes underlying brain aging and neurodegeneration. This know-how is crucial for developing new therapies and preventive strategies to alleviate the burden of these diseases on individuals, families, and healthcare systems.

**Material(s) and Method(s):**

Study Population: A balanced dataset of 2000 T1W-MPRAGE brain scans from ethnic Chinese individuals, stratified into 10-year age cohorts, was obtained at Sengkang General Hospital with informed consent and ethical approval. Scans were rigorously reviewed by three board-certified radiologists, and subjects with neurological conditions were excluded.

End-to-End Tool: The developed tool automates the analysis pipeline:

1. Data Conversion: DICOM files are standardized to BIDS-compliant NIfTI format.
2. Brain Segmentation: AssemblyNet deep learning model segments brain regions.
3. Template Creation: Segmented images are normalized to MNI space, generating 20 age- and gender-specific templates using ANTS (10 for each gender).
4. Volumetric Analysis: Absolute volumes, intracranial-normalized percentages, and left–right asymmetry are computed for each brain region.
5. Statistical Evaluation: Age-related changes are assessed using Student’s t-test, Wilcoxon, and Kruskal–Wallis tests.

Technical Implementation: The tool's front end is built with React, the backend utilizes Flask, and PostgreSQL manages the database. Docker ensures scalability and reproducibility of the system.

**Results:**

Our model accurately predicts brain age in a Singaporean ethnic Chinese cohort, capturing complex age-related volume changes, particularly in the 40–70 age range. Significant gender differences in cerebellar and cerebellum volumes were observed, emphasizing the need for tailored neurological assessments.

**Conclusions:**

This scalable and reproducible tool provides a comprehensive solution for analyzing brain aging, highlighting the importance of personalized interventions, enhance clinical applications, and advancing neuroimaging research by contributing to improved outcomes for individuals experiencing age-related cognitive decline and neurodegenerative diseases.

### P21 Voxel-Based Morphometric Analysis and Machine Learning Classification of Healthy Controls and Vascular Dementia Using MRI

#### Bhanu Prakash KN^1^, Kah Meng Ang^2^, Arvind CS^1^, Pulickal Geoiphy George^3^, Philip Yap Lin Kiat^4^, Cheong Chin Yee^4^, Julian Chieng Sau Lian^5^, Singh Dinesh Rambachan^3^

##### ^1^Clinical Data Analytics and Radiomics, Bioinformatics Institute, ASTAR, Singapore; ^2^Biomedical Engineering, National University of Singapore, Singapore; ^3^Department of Diagnostic Radiology, Khoo Teck Puat Hospital (KTPH), Singapore; ^4^Geriatric Medicine, Khoo Teck Puat Hospital (KTPH), Singapore; ^5^Department of Diagnostic Radiology, Woodlands Health, Singapore

*Cancer Imaging (2024),* **24 (1):** P21

**Objectives/ Teaching Points:**

Active ageing is markedly affected by dementia, a progressive neurodegenerative disorder impacting cognitive, functional, and behavioural domains, thereby diminishing the quality of life. Early diagnosis of dementia is crucial for timely interventions, which can alleviate healthcare burdens and significantly improve the well-being of affected individuals. This study employs magnetic resonance (MR) imaging and AI framework for voxel-based volumetry and classification of healthy controls (HC) and vascular dementia (VD).

**Material(s) and Method(s):**

This study included T1-weighted MPRAGE MRI scans from 200 HC and 70 individuals with VD from KTPH, with informed consent and ethical approval. The MR scans were anonymized, quality-checked using MRIQC, and arranged in BIDS format. Voxel-based morphometry was performed using AssemblyNet, and hippocampal volume was quantified using HippUnfold. The HC and VD groups were age and gender-matched with median ages of 63 and 66 years, respectively. Subcortical structural volumes, percentages and asymmetry were utilized as features for classification using Support Vector Machine (SVM), Random Forest (RF), and Logistic Regression (LR) classifiers, with GridSearchCV employed for hyperparameter optimization.

**Results:**

The SVM classifier performed best with absolute volume and asymmetry-based features, yielding an accuracy of 0.817, precision of 0.667, recall of 0.667, and an F1-score of 0.735. The RF classifier showed strong performance with volumetric percentage features. Subcortical structure-based features resulted in higher classification accuracy than lobe or cortical structures. Classification based on hippocampal features yielded an accuracy of 0.847, precision of 0.762, recall of 0.727, and an F1-score of 0.744.

**Conclusions:**

This study demonstrates the efficacy of combining advanced neuroimaging techniques with machine learning algorithms to differentiate between HC and individuals with VD. The nuanced differences in brain structures, particularly in the hippocampal subregions, highlight the potential of these methods for early and accurate diagnosis. The superior performance of AI-based segmentation and classification underscores their promise as powerful tools in clinical practice, paving the way for improved patient outcomes and more targeted therapeutic interventions. The integration of such technologies into routine diagnostic protocols could revolutionize the management of dementia, significantly enhancing the quality of life for affected individuals.

### P22 Errors in oncology CT: Analysis of a discrepancy database over 8 years

#### Kirsten Gormly, Kimberly Brown, Aanchal Agarwal

##### Radiology, Jones Radiology, Australia

*Cancer Imaging (2024),* **24 (1):** P22

**Objectives/ Teaching Points:**

To identify Oncology reporting misses or misinterpretations, use of oncology templates (OT) and any association between misses and OT reporting.

**Material(s) and Method(s):**

Retrospective audit of CT oncology cases in a radiology discrepancy database from 2014–2021 to identify location of misses, if typical of primary tumour, ease of visibility and potential clinical significance. Structure of the report was evaluated, in particular if an OT was used, measurements provided, conclusion terminology and any association between OT use and errors.

**Results:**

666 discrepancy cases included 134 oncology CT reports reported by 36 radiologists. 96% were detection misses. Most frequent missed lesions were bone (18%), lung (17%) and lymph nodes (13%), with 68% located at a typical site of spread, and considered easy to see in 38%, 43% and 65% respectively. 80% of errors had a likely effect on treatment.

92/134 reports used an OT. 16 radiologists always used a template and 7 never did. The conclusion contained RECIST terms in 31 and subjective descriptors in 33 reports. 68/69 cases with measurable lesions provided measurements and 22/69 used a table. No significant links between OT use and error rates were observed. However, there was a higher percentage of lymph node and lung misses without an OT.

**Conclusions:**

Detection misses were most common, with bone lesions, lung lesions and lymph nodes the most frequent, despite often being easy to see at typical sites of spread. Templates were frequently used with measurements included, but variable conclusions terms. No demonstrable link between OT use and misses.

### P23 Photon-counting detector CT in cardiovascular imaging

#### Mon Ben Chow, Jeffrey Seow Kuang Goh, Peter Yu-Tang Goh, Robert Khoon Kwok

##### Radiology, Parkway, Singapore

*Cancer Imaging (2024),* **24 (1):** P23

**Objectives/ Teaching Points:**

Cardiovascular diseases are the leading cause of death worldwide. CT imaging is a vital tool that provides crucial information for managing these conditions. The latest advancement in this technology is Photon-Counting Detector (PCD) CT. It offers significant improvements over the traditional Energy Integrating Detector (EID) CT. This poster will highlight the differences between PCD CT and EID CT, look into the technical details and terminology for PCD CT, offer protocol guidance, and showcase clinical applications through case examples.

### P24 The various facades of gallbladder carcinoma and its mimics

#### Salwa Al Sarahani, Mei Chin Lim

##### Department of Diagnostic Imaging, National University Hospital, Singapore

*Cancer Imaging (2024),* **24 (1):** P24

Gallbladder carcinoma is the most common cancer of the biliary system. Unfortunately, the gallbladder cancer is an aggressive tumour and typically presents late, resulting in poor prognosis. It can be challenging to diagnose gallbladder cancer because patients are often asymptomatic or present with non-specific signs and symptoms that may mimic other benign conditions such as chronic cholecystitis (including xanthogranulomatosis cholecystitis), cholesterol polyps, adenomyomatosis as well as other malignancy such as lymphoma, metastasis, or direct invasion of primary liver cancer. Furthermore, the risk factors for gallbladder cancer such as cholelithiasis also overlap with the other benign gallbladder conditions.

Early-stage tumours are often incidentally detected at imaging or during cholecystectomy performed for another indication. Typical imaging features of localized disease include asymmetric focal or diffuse thickening of the gallbladder wall, polyps larger than 1.0 cm or a solid mass replacing the gallbladder lumen. Advanced tumours are often infiltrative with adjacent organs invasion and nodal metastasis, and may even present with peritoneal disease. We aim to demonstrate cases of gallbladder carcinoma at different stages of disease as well as their mimics on cross-sectional imaging, providing imaging pearls that may help to suggest the diagnosis of gallbladder cancer.

### P25 Multi-parametric MRI Radiomics Model for Risk Stratification in Endometrial Carcinoma

#### Jiarui Zhang^1^, Rahul Singh^1^, Cheuk Nam Hwang^1^, Philip Ip^2^, Ka Yu Tse^3^, Elaine Yuen Phin Lee^1^

##### ^1^Department of Diagnostic Radiology, University of Hong Kong, Hong Kong; ^2^Department of Pathology, University of Hong Kong, Hong Kong; ^3^Department of Obstetrics and Gynaecology, University of Hong Kong, Hong Kong

*Cancer Imaging (2024),* **24 (1):** P25

**Objectives/ Teaching Points:**

Risk stratification in endometrial carcinoma (EC) determines the need for adjuvant treatment. We aimed to develop MRI radiomics model in preoperative risk stratification of EC.

**Material(s) and Method(s):**

This retrospective study collected MRI data of 238 patients with pathological confirmed EC from November 2018 to July 2023. Those with incomplete MRI, significant artefacts and no visible tumour on MRI were excluded. Patients were risk stratified based on the ESGO/ESTRO/ESP recommendation using the standard histopathological factors. Radiomics features were extracted from apparent diffusion coefficient (ADC) map (b = 0,400,800 s/mm2), T2-weighted imaging (T2WI) and dynamic contrast-enhanced (DCE)-MRI using PyRadiomics package (version 3.1). All patients were divided into training cohort and testing set with stratified ratio of 85:15. Highly correlated (> 90%) features were eliminated using spearman correlation and most significant features were selected employing rank-based feature selection approach known as Boruta with XGBoost as the estimator. ADASYN (Adaptive Synthetic) as applied to mitigate class imbalance. Pruned and hyper-parameter tuned Gradient Boosting classifier with tenfold stratified crossvalidation (SCV) was employed to develop the framework. The receiver operating characteristic (ROC) curves analysis and calibration curves were utilized to select and evaluate the best model and hyperparameters for risk stratification.

**Results:**

A total of 162 eligible patients were divided into 3 risk groups, 77 in the low-risk, 35 in the intermediate-risk and 50 in the high-risk groups. Seven features were selected (4 from ADC map, 2 from T2WI and 1 from DCE-MRI) in the multi-parametric radiomics models. The selected best model achieved the following performance metrics: area under the curve (0.801), sensitivity (0.778), specificity (0.870) and accuracy (0.760) in test set.

**Conclusions:**

The multi-parametric MRI-based radiomics model demonstrated favourable performance in preoperative risk stratification of EC, providing information on treatment stratification and prognosis.

### P26 Investigating the effect of contrast dilution on multiarterial phase image quality in gadoxetic acid-enhanced liver MRI

#### Jordan Zheng Ting Sim, Lee Chau Hung, Low Hsien Min

##### Diagnostic Radiology, Tan Tock Seng Hospital, Singapore

*Cancer Imaging (2024),* **24 (1):** P26

**Objectives/ Teaching Points:**

To assess the impact of dilution on multiarterial-phase image quality in liver magnetic resonance imaging (MRI) enhanced with gadoxetic acid (Gd-EOB-DTPA).

**Material(s) and Method(s):**

This retrospective study included a cohort of 81 patients who had serial gadoxetic acid-enhanced MRI of the liver, first with undiluted, then with 1:1 diluted contrast. The serial MRI examinations were done less than a year apart. Two expert readers independently rated the randomized multi-arterial phase images for anatomic conspicuity, respiratory motion artifacts and overall image quality using a five-point Likert scale. Statistical significance was tested with the Wilcoxon rank test at a significance level of P = 0.05.

**Results:**

The dilution of gadoxetic acid resulted in significantly better overall image quality (P = 0.04) although neither anatomic conspicuity nor respiratory artifacts showed significant improvement. The mean scores for anatomic conspicuity, respiratory artifacts and overall image quality were 3.72, 3.54 and 3.67 in the diluted group vs 3.59, 3.41 and 3.51 in the undiluted group respectively.

**Conclusions:**

An imaging protocol involving diluted gadoxetic acid improved overall image quality but do not seem to significantly reduce respiratory artifacts.

### P27 MRI-Based Investigation of Age-Related Hippocampal Changes in a Large Singaporean Cohort with Gender-Specific Effects

#### Bhanu Prakash KN^1^, Arvind CS^1^, Ashutosh Raj Shrestha^2^, Oliver James Nickalls^3^, Parag R. Salkade^3^, Ho Chi Long^3^

##### ^1^Clinical Data Analytics and Radiomics, Bioinformatics Institute, ASTAR, Singapore; ^2^Information Technology, Vellore Institute of Technology, India; ^3^Department of Radiology, Sengkang General Hospital, Duke-NUS Medical School, NUS Yong Loo Lin School of Medicine, Singapore

*Cancer Imaging (2024),* **24 (1):** P27

**Objectives/ Teaching Points:**

Hippocampal structural changes are linked to age-related cognitive decline, making their understanding crucial for addressing the challenges of Singapore's aging population. This study aimed to establish a population-specific normative reference for hippocampal volume changes with age, facilitating early detection of neurodegenerative diseases and enabling targeted interventions.

**Material(s) and Method(s):**

250 T1-weighted MPRAGE scans from ethnic Chinese individuals (50% male, 50% female) were obtained at Sengkang General Hospital, with ethical approval. Participants were stratified into age cohorts, excluding those with neurological conditions. Scans were reviewed by radiologists for quality assurance.

The BIDS-formatted data was analysed using Hippunfold, a state-of-the-art U-Net based automated hippocampal unfolding tool which facilitated precise morphometry and subfield segmentation, yielding volumetric measurements of both left and right CA1, CA2, CA3, CA4, Dentate gyrus (DG), subiculum (Sub), and a combined region encompassing Stratum, Radiatum, Lacunosum, and Moleculare (SRLM). To discern the significance of age-related changes in hippocampal structure, comprehensive statistical analyses Student's t-tests for parametric comparisons, and non-parametric Wilcoxon and Kruskal–Wallis tests to account for potential deviations from normality was conducted.

**Results:**

Hippocampal subfield volumes (CA1, CA2, CA3, CA4, Sub, and SRLM) followed similar trends in both hemispheres, with no gender differences in these patterns. Subfield volumes increased until age 30, then decreased for all subfields. Significant differences between genders were found for all subfields except DG in the 21–30 age group. Left and right subiculum volumes showed significant differences up to age 50.

**Conclusions:**

This study establishes a normative reference for age-related hippocampal subfield volume changes in Singapore's ethnic Chinese population. Consistent trajectories were observed, with volumes increasing until age 30, then declining. While generally no gender differences were found, subtle variations existed in specific subfields. These findings provide a crucial baseline for identifying deviations potentially indicating neurodegeneration, facilitating timely interventions, and improving the quality of life for Singapore's aging population.

### P28 Comparative analysis of computed tomography staging of colon cancer with postoperative histopathological correlation

#### Sneha Bhattacharya, Priya Ghosh, Sumit Mukhopadhyay, Saugata Sen, Anurima Patra, Anisha Gehani

##### Radiodiagnosis, Tata Medical Centre, India

*Cancer Imaging (2024),* **24 (1):** P28

**Objectives/ Teaching Points:**

CT is the modality of choice for staging colon cancers. Neoadjuvant therapies are established in rectal cancer management, but the standard treatment for colon cancer has been upfront surgery. Trials such as FOxTROT are establishing the role of neoadjuvant therapies in downstaging locally advanced colon cancers (T3-T4 stage), thus emphasising the role of preoperative CT staging. This retrospective study aims to compare preoperative CT-based T-staging with postoperative histopathological T-staging in patients undergoing upfront surgery for biopsy-proven colon adenocarcinoma. Secondary objective includes correlating locoregional lymphadenopathy.

**Material(s) and Method(s):**

Adult patients undergoing upfront colectomy for biopsy-proven colon adenocarcinoma between Jan-July 2022 with adequate preoperative CT abdomen, were selected. Patients with metastases, colon perforation and intussusception, were excluded. T and N staging was done by an experienced radiologist on preoperative CT, and correlation was done with post-operative histopathology using appropriate statistical methods.

**Results:**

51 patients (28 females, 23 males) were included in the study. According to radiological staging, the numbers of T2, T3, T4a and T4b cancers were 17, 25, 5 and 4 respectively. CT T stage was concordant with pathology T stage in 52.9% cases. For detecting tumour invasion beyond the bowel wall (T3, T4), CT showed pooled sensitivity and specificity were 68% (53–81%) and 50% (7–93%), respectively. Positive predictive value (PPV) (95%CI) was 94% (85–98%), but negative predictive value was 12% (4–28%). For nodal involvement (N +), sensitivity was 72% (47–90%) and specificity was 68% (38–88%).

**Conclusions:**

CT showed high PPV for detecting colon wall invasion in locally advanced colon cancers, but the sensitivity and specificity of T and N-staging were modest. Our findings highlight the value of CT for preoperative cancer staging, which can potentially guide the selection of candidates for neoadjuvant therapy in colon cancer.

### P29 Comprehensive Imaging of Advanced Ovarian Carcinoma: Clinicoradiological Correlation and Prognostic Value of Peritoneal Carcinomatosis Index—Perspectives from an Oncology Institute

#### Akshat Shah, Priya Ghosh, Saugata Sen

##### Department of Radiology and Imaging, Tata Medical Center—Cancer Hospital and Research Center, Kolkata, West Bengal, India

*Cancer Imaging (2024),* **24 (1):** P29

**Objectives/ Teaching Points:**

This study aims to reveal imaging features of advanced ovarian carcinoma with special focus on Peritoneal Carcinomatosis Index (PCI) with its prognostic significance.

**Material(s) and Method(s):**

We conducted a retrospective analysis of patients diagnosed with advanced ovarian carcinoma who underwent imaging assessments (CT and PET-CT) and treatment (upfront debulking surgery / interval standard/advanced debulking surgery/HIPEC) at our institute from October 2011 to March 2024.

Detailed analysis of varied appearance of imaging findings of advanced ovarian carcinoma was done. Clinicoradiological correlation was established by comparing preoperative computed tomography (CT) and CT-PCI findings with intraoperative PCI scores. The prognostic value of PCI was determined by analysing its association with disease progression and patient outcomes.

**Results:**

The study cohort comprised 220 patients exhibiting diverse presentations of advanced ovarian carcinoma on CT scans, demonstrating involvement of the peritoneum, omentum, and mesentery, with lesion sizes spanning 1 to 3. A robust correlation (Pearson correlation coefficient – 0.9, p < 0.001) was observed between preoperative CT PCI scores and operative PCI scores. Elevated PCI scores significantly correlated with increased disease progression on follow-up scans in form of omental, peritoneal, organ and bony metastatic lesions. Lower PCI scores correlates with better prognosis and disease free interval/survival. Furthermore, the analysis revealed high specificity and sensitivity of the CT-PCI scoring system in predicting intra-abdominal disease burden, with specificity of 90% and sensitivity of 85% in detecting advanced disease stages.

**Conclusions:**

Clinicoradiological correlation is vital in determining disease extent and guiding treatment strategies for advanced ovarian carcinoma. The strong relationship between preoperative CT PCI and intraoperative PCI underscores imaging techniques' dependability in predicting intra-abdominal disease involvement. PCI serves as a valuable prognostic indicator, aiding in risk stratification and disease monitoring. Assessing PCI's influence on treatment strategies optimizes therapy. Analyzing CT imaging findings enhances its predictive value, while long-term follow-up tracks PCI score changes and disease prognosis.

### P30 Paediatric Liver Masses: An imaging spectrum

#### Vanessa Fernandes

##### Radiology, Kidwai Memorial Institute of Oncology, India

*Cancer Imaging (2024),* **24 (1):** P30

**Objectives/ Teaching Points:**

To illustrate radiologic presentations of paediatric liver masses and their mimics.

**Material(s) and Method(s):**

We reviewed seven paediatric cases retrospectively presenting with liver masses at over the last year at our institution. The cases included a spectrum of pathologies: benign and malignant neoplasms and mimicks. All patients underwent a combination of imaging modalities supplemented by histopathological examination where necessary.

**Results:**

- Infantile Hepatic Haemangioma: A 2-month old infant with a large, well defined hyperechoic lesion on US, confirmed by CT to be haemangioma.
- Hepatoblastoma: A 3-year old with a rapidly enlarging abdominal mass, showing a heterogeneous lesion with calcifications on CT, confirmed by biopsy as hepatoblastoma.
- Focal Nodular Hyperplasia (FNH): A 9-year old with an incidental finding on US, further characterized by MRI showing a central scar, indicative of FNH.
- Fibrolamellar Hepatocellular Carcinoma (HCC): A 14-year old with chronic liver disease, presenting with a hypervascular lesion on CT, consistent with fibrolamellar HCC on biopsy.
- Embryonal Sarcoma: A 6-year old with a large, cystic mass with solid components on CT, diagnosed as embryonal sarcoma post-surgery.
- Tuberculomas: An 8-year old with multiple complex cystic lesion on US and CECT diagnosed as multifocal hepatoblastoma confirmed after biopsy to be tuberculosis.
- Fungal Abscess: A 5-year old with congenital neutropenia with a liver lesion detected on USG, further on CT demonstrating a hyperdense lesion showing rim-enhancement.
- Radiological evaluation, particularly through the integration of US, CT, and MRI, was pivotal in differentiating among various liver masses and their mimics.

**Conclusions:**

This case series underscores the critical role of radiological imaging in the evaluation of paediatric liver masses. A multimodal imaging approach helps in distinguishing between benign, malignant, and non-neoplastic mimics, thus optimizing patient management and outcomes.

